# Anti-HIV-1 Activity of Lactic Acid in Human Cervicovaginal Fluid

**DOI:** 10.1128/mSphere.00055-18

**Published:** 2018-07-05

**Authors:** David Tyssen, Ying-Ying Wang, Joshua A. Hayward, Paul A. Agius, Kevin DeLong, Muriel Aldunate, Jacques Ravel, Thomas R. Moench, Richard A. Cone, Gilda Tachedjian

**Affiliations:** a Disease Elimination Program, Life Sciences Discipline, Burnet Institute, Melbourne, Victoria, Australia; b Department of Biophysics, Johns Hopkins University, Baltimore, Maryland, USA; c Maternal and Child Health Program, Public Health Discipline, Burnet Institute, Melbourne, Victoria, Australia; d Department of Epidemiology and Preventative Medicine, Monash University, Melbourne, Victoria, Australia; e Department of Biology, Johns Hopkins University, Baltimore, Maryland, USA; f Department of Microbiology, Monash University, Clayton, Victoria, Australia; g Institute for Genome Sciences, University of Maryland School of Medicine, Baltimore, Maryland, USA; h Department of Microbiology and Immunology, University of Maryland School of Medicine, Baltimore, Maryland, USA; i Mapp Biopharmaceutical, San Diego, California, USA; j Department of Microbiology and Immunology, University of Melbourne, at the Peter Doherty Institute for Infection and Immunity, Melbourne, Victoria, Australia; k School of Science, College of Science, Engineering and Health, RMIT University, Melbourne, Victoria, Australia; University of Florida

**Keywords:** HIV transmission, *Lactobacillus*, human immunodeficiency virus, metabolite, microbiota, vagina

## Abstract

Women of reproductive age with a *Lactobacillus-*dominated vaginal microbiota have a reduced risk of acquiring and transmitting HIV and a vaginal pH of ~4 due to the presence of ~1% (wt/vol) lactic acid. While lactic acid has potent HIV virucidal activity *in vitro*, whether lactic acid present in the vaginal lumen inactivates HIV has not been investigated. Here we evaluated the anti-HIV-1 activity of native, minimally diluted cervicovaginal fluid obtained from women of reproductive age (*n* = 20) with vaginal microbiota dominated by *Lactobacillus* spp. Inhibition of HIV_Ba-L_ was significantly associated with the protonated form of lactic acid in cervicovaginal fluid. The HIV_Ba-L_ inhibitory activity observed in the <3-kDa acidic filtrate was similar to that of the corresponding untreated native cervicovaginal fluid as well as that of clarified neat cervicovaginal fluid subjected to protease digestion. These *ex vivo* studies indicate that protonated lactic acid is a major anti-HIV-1 metabolite present in acidic cervicovaginal fluid, suggesting a potential role in reducing HIV transmission by inactivating virus introduced or shed into the cervicovaginal lumen.

**IMPORTANCE** The *Lactobacillus*-dominated vaginal microbiota is associated with a reduced risk of acquiring and transmitting HIV and other sexually transmitted infections (STIs). Lactic acid is a major organic acid metabolite produced by lactobacilli that acidifies the vagina and has been reported to have inhibitory activity *in vitro* against bacterial, protozoan, and viral STIs, including HIV infections. However, the anti-HIV properties of lactic acid in native vaginal lumen fluids of women colonized with *Lactobacillus* spp. have not yet been established. Our study, using native cervicovaginal fluid from women, found that potent and irreversible anti-HIV-1 activity is significantly associated with the concentration of the protonated (acidic, uncharged) form of lactic acid. This work advances our understanding of the mechanisms by which vaginal microbiota modulate HIV susceptibility and could lead to novel strategies to prevent women from acquiring HIV or transmitting the virus during vaginal intercourse and vaginal birth.

## Abstract (precis)

The *Lactobacillus*-dominated vaginal microbiota is associated with a reduced risk of acquiring and transmitting HIV and other sexually transmitted infections (STIs). Lactic acid is a major organic acid metabolite produced by lactobacilli that acidifies the vagina and has been reported to have inhibitory activity *in vitro* against bacterial, protozoan, and viral STIs, including HIV infections. However, the anti-HIV properties of lactic acid in native vaginal lumen fluids of women colonized with *Lactobacillus* spp. have not yet been established. Our study, using native cervicovaginal fluid from women, found that potent and irreversible anti-HIV-1 activity is significantly associated with the concentration of the protonated (acidic, uncharged) form of lactic acid. This work advances our understanding of the mechanisms by which vaginal microbiota modulate HIV susceptibility and could lead to novel strategies to prevent women from acquiring HIV or transmitting the virus during vaginal intercourse and vaginal birth.

## INTRODUCTION

Human immunodeficiency virus (HIV) is primarily transmitted by sexual contact and can establish infection via entry through the genital mucosa (1, 2). Variation in the probability of HIV acquisition or transmission may be explained in part by differences in the vaginal microbiota (3). Vaginal colonization with Lactobacillus crispatus is associated with reduced risk in women of acquiring HIV (4, 5). HIV-infected women with microbiota dominated by *Lactobacillus* spp., particularly L. crispatus, have a lower risk of HIV genital shedding, which is thought to be associated with reduced HIV transmission to their sexual partners or neonates during vaginal birth (5–11). In contrast, women with vaginal microbiota characterized by a high diversity of anaerobes (e.g., *Gardnerella*, *Prevotella*, and *Atopobium* spp.) and a paucity of vaginal lactobacilli, as exemplified in cases of bacterial vaginosis (BV) (12), are at higher risk of acquiring HIV from (13, 14) and transmitting HIV to (15) their male partners. A high relative abundance of *Lactobacillus* spp. in the vagina is also associated with a lower risk of developing BV and of acquiring sexually transmitted infections (STIs), including genital herpes, chlamydia, and gonorrhea, and with other more favorable reproductive health outcomes compared to women lacking *Lactobacillus* spp. (16, 17).

Vaginal *Lactobacillus* spp. are thought to help protect against pathogens principally through the secretion of antimicrobial factors, such as bacteriocins and organic acid metabolites (16, 18, 19). The major organic acid metabolite produced by *Lactobacillus*-dominated microbiota is lactic acid (LA), defined here as the sum of the charged lactate anion and the protonated lactic acid. *Lactobacillus* spp. produce l- and d-isomers of lactic acid, and the ratio of these isomers in the vagina is characteristic of *Lactobacillus* spp. colonizing the vagina (20, 21), indicating that the majority of vaginal lactic acid is produced by lactobacilli (19). When the vagina of a reproduction-age woman is colonized with *Lactobacillus* spp., lactic acid levels within the native cervicovaginal fluid (CVF; i.e., undiluted and containing host cells, bacteria, proteins, and mucin-associated glycoproteins) average 1.0% ± 0.2% (wt/vol) (~110 mM) (22). Lactic acid drives the acidification of the vagina (20, 21) to a pH of 3.5 ± 0.3 (measured under the hypoxic and hypercapnic conditions of the vaginal lumen) and is the dominant buffer below pH 4.2 (22). In contrast, women lacking *Lactobacillus* spp., such as found in those with BV, have dramatically reduced levels of lactic acid (23–25) and elevated vaginal pH levels of >4.5 (12). These observations, together with the epidemiological evidence indicating that a *Lactobacillus*-dominated microbiota reduces a woman’s risk of acquiring or transmitting HIV (13–15), suggest that lactic acid represents a critical antimicrobial defense factor in the vagina.

We and others have previously shown that lactic acid has *in vitro* inhibitory activity against bacterial, protozoan, and viral STIs, including HIV (26–32). Our previous *in vitro* study demonstrated that lactic acid has broad-spectrum virucidal activity against HIV type 1 (HIV-1) and type 2 (HIV-2) and against various HIV subtypes as well as against transmitted/founder strains of HIV-1 (31). Lactic acid at physiological concentrations and pH potently and rapidly inactivates HIV-1 in a concentration- and pH-dependent manner (31). HIV-1 inactivation in the presence of lactic acid is dramatically more potent than in the presence of low pH alone (acidified by HCl) or in the presence of acetic acid, another organic acid that is small in size and found at higher levels in the vagina of women with BV (31). Lactic acid is in equilibrium between two states defined by the presence (protonated lactic acid) or the absence (lactate anion) of a hydrogen atom on the carboxylic acid as described by Aldunate et al. (16). The lactate anion, heavily dominant at neutral pH, is devoid of HIV-1 virucidal activity, suggesting that protonated lactic acid, predominating at pH levels below the 3.86 pK_a_ of lactic acid, mediates HIV-1 inactivation (31). While our *in vitro* studies demonstrated that lactic acid at low pH (and thus predominantly protonated) is a highly potent anti-HIV metabolite, no studies have examined whether protonated lactic acid inactivates HIV-1 in native CVF.

The present study is designed to assess whether CVF from women of reproductive age inactivates HIV-1 *ex vivo* and whether the protonated lactic acid concentration in CVF is associated with HIV-1 inactivation potency. A strong association would suggest that protonated lactic acid in the CVF of women with a *Lactobacillus*-dominated vaginal microbiota is capable of inactivating HIV-1 and thus may contribute to protection against HIV-1 acquisition or transmission during vaginal intercourse and vaginal birth.

## RESULTS

### Demographic characteristics of study participants.

A total of 20 women were recruited from staff and students at Johns Hopkins University; the majority were young (mean age ± SD, 23 ± 2.9; *n =* 19) (see Table S1 in the supplemental material). Most of the participants identified as non-Hispanic white (*n =* 12), with the remainder identifying as Asian (*n =* 4), black (*n =* 1), other (*n =* 1), and ethnicity data not available (*n =* 2) (Table S1). A total of 23 CVF samples were collected, with 3 of the 20 women (participants 2, 3, and 4) donating 2 samples each (Table S1). Most of the tested CVF samples (22/23) were assigned a Nugent score of 0 to 3, indicative of *Lactobacillus*-dominated microbiota, while one sample (HIV_25) was intermediate (Nugent score of 5) (Table S2). CVF collected on separate days from 2 of 3 women (participants 2 and 4) (Table S1) with available Nugent scores for both samples were non-BV (Nugent score 0 to 3) (Table S2). HIV_18, the single sample from participant 3 with Nugent data, was also non-BV (Table S2).

10.1128/mSphere.00055-18.2 TABLE S1  Donor demographic data. Download TABLE S1, PDF file, 0.1 MB. Copyright © 2018 Tyssen et al. 2018 Tyssen et al. This content is distributed under the terms of the Creative Commons Attribution 4.0 International license.

10.1128/mSphere.00055-18.3 TABLE S2  Donor sample characterization. Download TABLE S2, DOCX file, 0.1 MB. Copyright © 2018 Tyssen et al. 2018 Tyssen et al. This content is distributed under the terms of the Creative Commons Attribution 4.0 International license.

### Native CVF has pH-dependent HIV-1 inhibitory activity.

**FIG 1:**
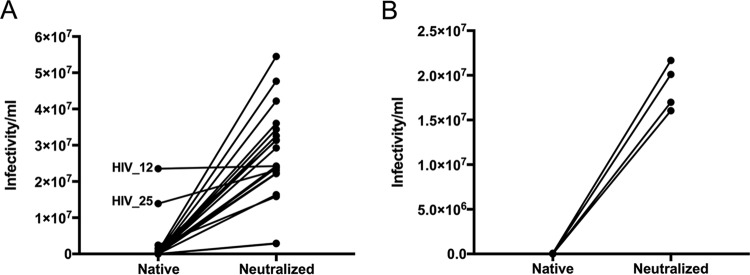
Most native, minimally diluted CVF samples fully inactivate HIV_Ba-L_ (A) and the transmitted/founder HIV_RHPA_ strain (B); however, inactivation is lost upon neutralization of CVF. Lines connect HIV_Ba-L_ and HIV_RHPA_ infectivity measurements from native and neutralized aliquots of the same CVF sample. HIV_12 and HIV_25 CVF samples are indicated. Data represent CVF samples from 22 distinct samples (from *n =* 20 women) (A) and 4 distinct samples (from *n =* 4 women) (B). Note that data points overlay for several samples in panel A.

To determine the HIV-1 inhibitory activity of native CVF, samples collected using a menstrual Softcup (33) were incubated with high-titer HIV_Ba-L_ (Fig. 1A) or HIV_RHPA_ (Fig. 1B) and assayed for infectivity using TZM-bl indicator cells. HIV_Ba-L_ is a CCR5-using (R5) HIV-1 laboratory strain, and HIV_RHPA_ is an R5 transmitted/founder strain (34). We observed significantly increased HIV_Ba-L_ infectivity per milliliter in neutralized CVF samples compared to native samples (*P* = 0.001, *n =* 22) (Table 1). Twelve samples showed HIV_Ba-L_ infectivity reduced to levels below the detection limit of the assay, while the majority of the other samples showed HIV_Ba-L_ infectivity that was reduced by 6-fold to 14,000-fold compared to HIV_Ba-L_ infectivity in separate aliquots of the same CVF samples adjusted to pH 7 with minimal dilution during neutralization. The exceptions were two samples (HIV_12 and HIV_25) with native pH values of 5.43 and 4.95, respectively, compared to pH ≤4.5 for all other samples (Table S2). The Nugent scores for HIV_12 and HIV_25 were 2 and 5, respectively, differing from the majority of samples with a Nugent score of 0 (Table S2).

**TABLE 1:** Association between HIV_Ba-L_ infectivity and HIV_RHPA_ infectivity (in milliliters) in neutralized CVF relative to native CVF from GLMM^a^

Virus and CVF category	*b* (SE)^b^	95% CI^c^	*P* value^d^
HIV_Ba-L_			
Native	Ref		
Neutralized	2.59 (0.79)	1.04, 4.14	0.001
			
HIV_RHPA_			
Native	Ref		
Neutralized	6.80 (0.76)	5.31, 8.28	<0.001

a Data represent generalized linear mixed modeling (GLMM) results generalized through specification of a Poisson distribution and log-link function from *n =* 44 data points for HIV_Ba-L_ and *n =* 8 data points for HIV_RHPA_. GLMM was used to apply a random intercept (gamma distribution assumed) for data from each study participant given the repeated HIV-1 infectivity measurement per participant. The (natural) coefficient is the log incidence rate ratio—an exponentiated coefficient yields the percentage of change for a unit (percent) increase for a given factor. The effect represents the difference in log incidence values between study participants’ native and neutralized CVF samples with each participant’s native CVF sample treated as the reference (Ref).

b Data represent log incidence rate ratio (*b*) values and associated standard errors (SE). Bootstrapped standard errors (*n =* 1,000 replications) were estimated to provide correct inferences in the presence of overdispersion in infectivity in generalized linear modeling.

c 95% CI, 95% confidence interval.

d *P* value, probability value. Statistical significance was determined at a *P* value of <0.01.

We also observed a significant increase in HIV_RHPA_ infectivity per milliliter in neutralized CVF samples compared to native samples (*P* < 0.001, *n =* 4) (Table 1). Three of the four native CVF samples (HIV_17, HIV_21, and HIV_22) showed HIV_RHPA_ infectivity that was reduced to below the detection limit of the assay compared to the corresponding neutralized CVF sample (Fig. 1B). These same CVF samples also demonstrated complete inactivation of HIV_Ba-L_ (Fig. 1A). A fourth CVF sample (HIV_4), unique to the HIV_RHPA_ experiments, demonstrated 270-fold inactivation of HIV_RHPA_ (Fig. 1B). Taken together, these data indicate that native CVF from the majority of women in our cohort possessed dramatic pH-dependent inhibitory activity against an R5 laboratory strain and an R5 transmitted/founder strain that was abrogated at neutral pH.

### Anti-HIV_Ba-L_ activity in CVF is independently associated with percent d+l-protonated lactic acid but not with percent d+l-lactate anion or pH.

**FIG 2:**
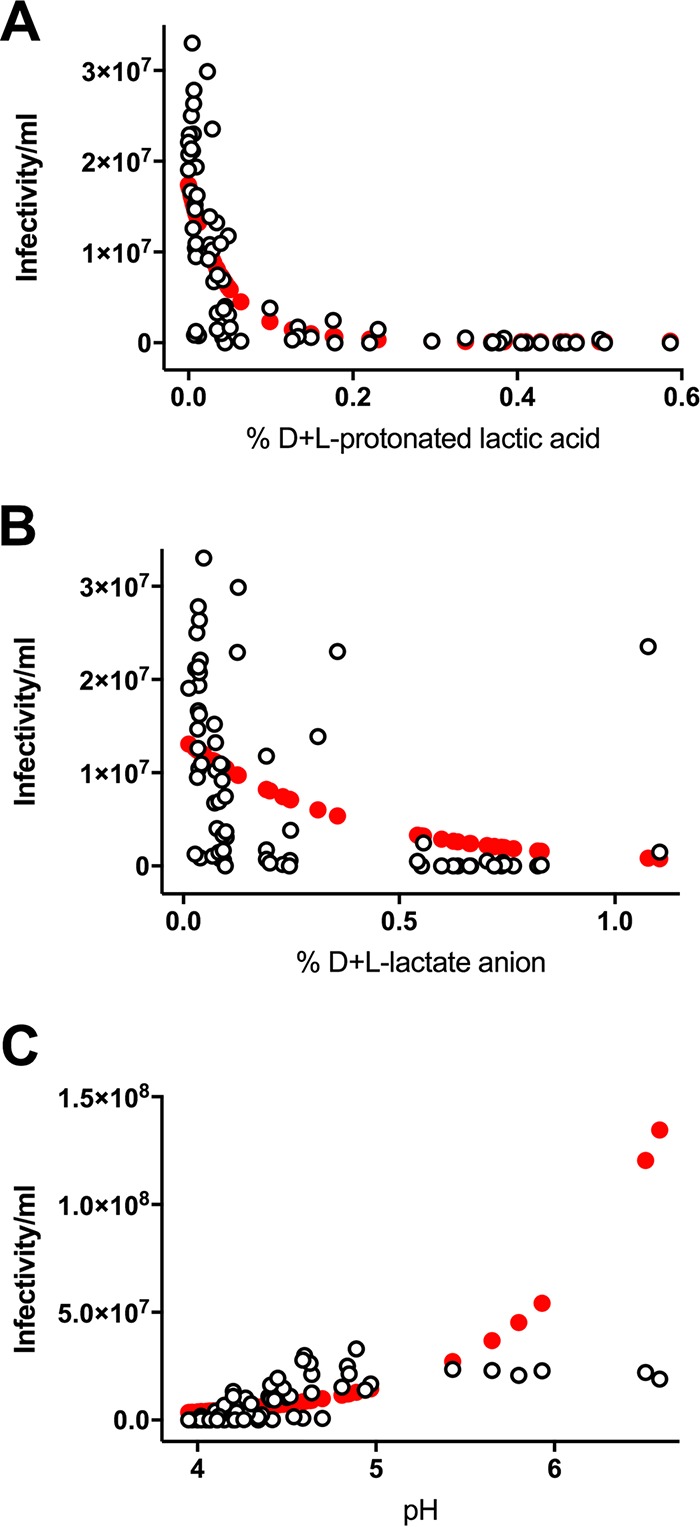
HIV_Ba-L_ inactivation in CVF is significantly associated with percent d+l-protonated lactic acid but not with percent d+l-lactate anion or with pH. Generalized linear mixed modeling (GLMM) was used to estimate the association between HIV_Ba-L_ infectivity per milliliter and CVF sample percent d+l-protonated lactic acid (A), percent d+l-lactate anion (B), and pH (C). Observed data from native, minimally diluted, 3-fold diluted, 9-fold diluted, 27-fold diluted, and neutralized CVF samples are depicted by open black circles and predicted values in filled red circles. Given the discrete (nonnormal) nature of the HIV_Ba-L_ infectivity outcome data, linear modeling was generalized by a Poisson distribution and log-link function. To account for inherent dependency in the data from repeated measures of HIV_Ba-L_ infectivity after CVF treatment, GLMM analyses specified a participant-specific random intercept effectively modeling the data corresponding to the heterogeneity between participants in person-specific HIV_Ba-L_ infectivity levels which induce correlation across serial measurements. Modeling data from unadjusted (univariable) analyses are shown where associations reached statistical significance for infectivity per milliliter versus percent d+l-protonated lactic acid (*P* < 0.001) but not versus percent d+l-lactate anion (*P* = 0.280) or pH (*P* = 0.139). Data represent CVF samples from 22 distinct samples (from *n =* 20 women).

**FIG 3:**
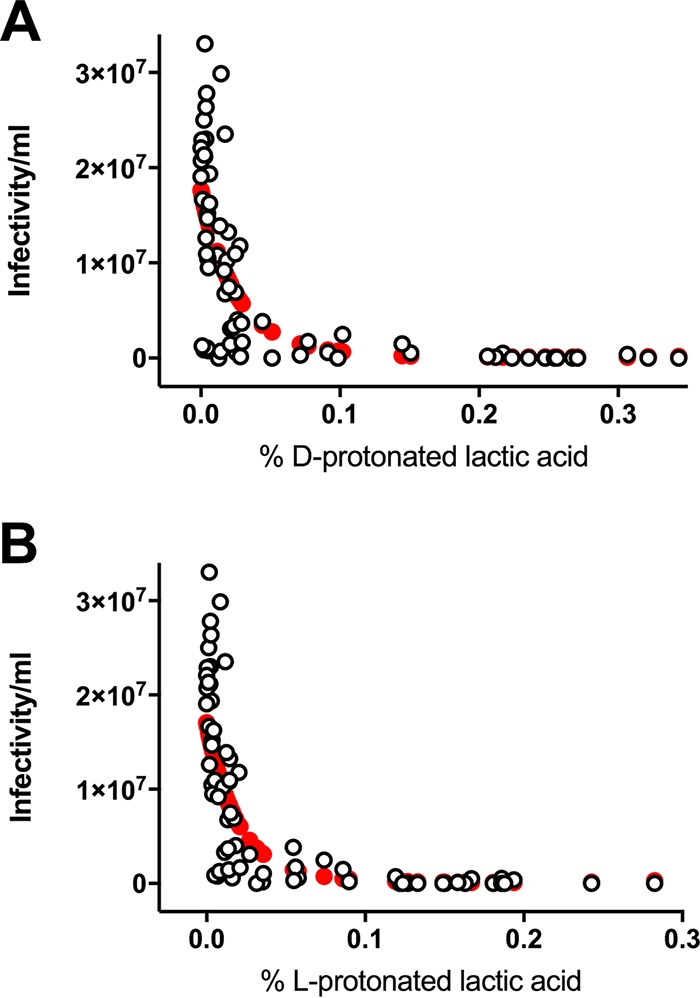
HIV_Ba-L_ inactivation is significantly associated with both percent d-protonated lactic acid and percent l-protonated lactic acid. Generalized linear mixed modeling (GLMM) was used to estimate the association between HIV_Ba-L_ infectivity per milliliter and CVF sample percent l-protonated lactic acid (A) and percent d-protonated lactic acid (B). Observed data from native, minimally diluted, 3-fold diluted, 9-fold diluted, 27-fold diluted, and neutralized CVF samples are depicted by open black circles and predicted values in filled red circles. Given the discrete (nonnormal) nature of the HIV_Ba-L_ infectivity outcome data, linear modeling was generalized by a Poisson distribution and log-link function. To account for inherent dependency in the data from repeated measures of HIV_Ba-L_ infectivity after CVF treatment, GLMM analyses specified a participant-specific random intercept effectively modeling the data corresponding to the heterogeneity between participants in person-specific HIV_Ba-L_ infectivity levels which induce correlation across serial measurements. Modeling data from unadjusted (univariable) analyses are shown where associations reached statistical significance for infectivity per milliliter versus percent l-protonated lactic acid (*P* < 0.001) and percent d-protonated lactic acid (*P* < 0.001). Data represent CVF samples from 22 distinct samples (from *n =* 20 women).

To investigate whether protonated lactic acid contributes to the HIV-1 inhibitory activity of native CVF *ex vivo*, we determined the association between anti-HIV_Ba-L_ activity in CVF and the protonated lactic acid levels in the samples (Fig. 2A). Similar levels of HIV-1 inactivation in the same CVF samples were observed for HIV_Ba-L_ and HIV_RHPA_ (Fig. 1). Accordingly, we performed subsequent experiments with HIV_Ba-L_ since we were able to generate 2-fold-higher titers of this virus, providing a greater dynamic range for our experiments. Since more than half of the samples showed HIV_Ba-L_ infectivity that was reduced to below the detection limit of the assay, we prepared aliquots of the same CVF samples diluted 1:3, 1:9, and 1:27 with unbuffered 0.9% saline solution to enable quantitation of HIV-1 infectivity in the samples over a range of titers. We used generalized linear mixed modeling (GLMM) to estimate the association between the measured level of HIV_Ba-L_ infectivity per milliliter and the percentage of d+l-protonated lactic acid in the CVF samples (Fig. 2A). Unadjusted (univariable) modeling analyses showed a significant association for HIV_Ba-L_ infectivity per milliliter with percent d+l-protonated lactic acid [Wald χ^2^_(2)_ = 107.8, *P* < 0.001] (Fig. 2A; see also Table 2). The association was such that an increase in percent d+l-protonated lactic acid resulted in a decrease in the level of HIV_Ba-L_ infectivity per milliliter; however, the more the magnitude of this decrease in infectivity declined, the greater the percentage of d+l-protonated lactic acid (Fig. 2A). A 0.1% increase in the proportion of d+l-protonated lactic acid yielded an approximately 7.6-fold decrease in HIV_Ba-L_ infectivity per milliliter, while a 0.5% increase in the percentage of d+l-protonated lactic acid yielded an approximate 156-fold decrease. This was calculated by exponentiation of the sum of products from the linear log incidence rate ratio (*b* = −22.8) and squared log incidence rate ratio (*b* = 25.5) at these levels of percent d+l-protonated lactic acid concentration (Table 2). Anti-HIV_Ba-L_ activity in CVF was also negatively associated with increasing levels of each of the protonated lactic acid isomers with respect to the percentage of d-protonated lactic acid [Wald χ^2^_(2)_ = 100.3, *P* < 0.001] and the percentage of l-protonated lactic acid [Wald χ^2^_(2)_ = 83.4, *P* < 0.001]. However, attenuations of these negative effects were also observed for each of the protonated lactic acid isomers as their levels increased [percent d-protonated lactic acid, Wald χ^2^_(1)_ = 9.32, *P* = 0.002; percent l-protonated lactic acid, Wald χ^2^_(1)_ = 10.2, *P* = 0.001] (Fig. 3; see also Table 3). In contrast, our unadjusted modeling analysis revealed no significant association between HIV_Ba-L_ infectivity and the percentage of d+l-lactate anion [Wald χ^2^_(1)_ = 1.2, *P* = 0.280] (Fig. 2B) or pH [Wald χ^2^_(1)_ = 2.2, *P* = 0.139] (Fig. 2C) present in CVF samples (Table 2).

**TABLE 2:** Unadjusted and adjusted associations between HIV_Ba-L_ infectivity and protonated d+l-lactic acid, d+l-lactate anion and pH from generalized linear mixed modeling^a^

Factor	Unadjusted			Model A^b^			Model B^c^			Model C^d^		
	*b* (SE)^e^	95% CI^f^	*P* value^g^	*b* (SE)	95% CI	*P* value	*b* (SE)	95% CI	*P* value	*b* (SE)	95% CI	*P* value
% d+l-protonated lactic acid												
Linear term	−22.8 (3.9)	−30.6, −15.1	<0.001	−24.6 (4.9)	−34.3, −14.9	<0.001	−27.9 (5.6)	−39.0, −16.9	<0.001	−26.9 (5.4)	−37.7, −16.3	<0.001
Squared term	25.5 (8.7)	8.48, 42.6	0.003	29.1 (10.3)	8.8, 49.4	0.005	33.4 (8.5)	16.8, 50.0	<0.001	31.2 (10.1)	11.4, 51.1	0.002
												
pH												
Linear term	1.38 (0.93)	−0.45, 3.20	0.139	−0.14 (0.35)	−0.83, 0.54	0.681				0.12 (0.22)	−0.30, 0.54	0.589
												
% d+l-lactate												
Linear term	−2.59 (2.4)	−7.29, 2.11	0.280				0.91 (1.6)	16.4, 16.9	0.573	0.98 (1.5)	−1.95, 3.92	0.511

a Data represent generalized linear mixed modeling (GLMM) results generalized through specification of a Poisson distribution and log-link function from *n =* 69 data points. GLMM was used to apply a random intercept (gamma distribution assumed) for data from each study participant given the repeated HIV-1 infectivity measurement per participant. HIV infectivity data were subjected to regression for each factor by applying either linear or quadratic functional forms. The (natural) coefficient is the log incidence rate ratio—exponentiated coefficients yield the percentage of change for a unit (percent) increase for a given factor.

b Model A data represent independent effects for percent d+l-protonated lactic acid and pH.

c Model B data represent independent effects for percent d+l-protonated lactic acid and percent d+l-lactate.

d Model C data represent independent effects for percent d+l-protonated lactic acid, pH, and percent d+l-lactate.

e Data represent log incidence rate ratio (*b*) values and associated standard errors (SE). Bootstrapped standard errors (*n =* 1,000 replications) were estimated to provide correct inferences in the presence of overdispersion in infectivity in generalized linear modeling.

f 95% CI, 95% confidence interval.

g *P* value, probability value. Statistical significance was determined at a *P* value of <0.01.

**TABLE 3:** Associations between HIV_Ba-L_ infectivity and d- and l-protonated lactic acid isomers

Factor^a^	*b* (SE)^b^	95% CI^c^	*P* value^d^
d-Protonated lactic acid			
Linear term	−40.3 (7.0)	−54.0, −26.5	<0.001
Squared term	77.3 (25.3)	27.7, 126.8	0.002
			
l-Protonated lactic acid			
Linear term	−52.4 (8.7)	−69.4, −35.4	<0.001
Squared term	134.3 (42.1)	51.8, 216.8	0.001

a Effects for each factor represent separate generalized linear mixed modeling (GLMM) analyses from *n =* 69 data points where infectivity data were subjected to regression for percent d-protonated lactic acid and percent l-protonated lactic acid applying a quadratic functional form and assuming a Poisson distribution and log-link function. GLMM was used to apply a random intercept (gamma distribution assumed) for data from each study participant given the repeated HIV-1 infectivity measurement per participant.

b Data represent log incidence rate ratio (*b*) value and associated standard errors (SE). Bootstrapped standard errors (*n =* 1,000 replications) were estimated to provide correct inference in the presence of overdispersion in infectivity in generalized linear modeling.

c 95% CI, 95% confidence interval.

d Statistical significance was determined at a *P* value of <0.01.

We next determined whether the anti-HIV_Ba-L_ activity of d+l-protonated lactic acid in CVF is independent of pH, of d+l-lactate anion, and of pH and d+l-lactate anion by performing multivariable analyses and applying three models A, B, and C, respectively (Table 2). Our generalized linear mixed modeling analyses revealed that percent d+l-protonated lactic acid remained significantly associated with anti-HIV_Ba-L_ in all three models even accounting for pH and/or the lactate anion [for model A, Wald χ^2^_(2)_ = 84.8, *P* < 0.001; for model B, Wald χ^2^_(2)_ = 24.9, *P* < 0.001; for model C, Wald χ^2^_(2)_ = 27.7, *P* < 0.001]. Collectively, these data demonstrate a strong and significant independent association between HIV_Ba-L_ infectivity in CVF and the protonated levels of d+l-lactic acid.

### pH-dependent anti-HIV-1 activity in CVF was predominately seen in the 3-kDa filtrate and was retained following pepsin digestion.

Intrinsic HIV inhibitory activity in CVF has been ascribed to the presence of cationic antimicrobial peptides (AMPs) that inhibit early stages of HIV-1 replication (35, 36). Most of these peptides have a molecular weight (MW) of >3 kDa (36). Thus, to differentiate the AMP HIV-1 inhibitory activity present in CVF from that of protonated lactic acid, pooled native CVF samples with high lactic acid levels ranging from 1.4% to 1.7% (wt/vol) were subjected to centrifugation through a 3-kDa molecular weight cutoff (MWCO) membrane to collect the <3-kDa filtrate. The smaller (90-Da) lactic acid is expected to partition into the <3-kDa filtrate, while most of the AMPs would be present in the ≥3-kDa fraction.

**FIG 4:**
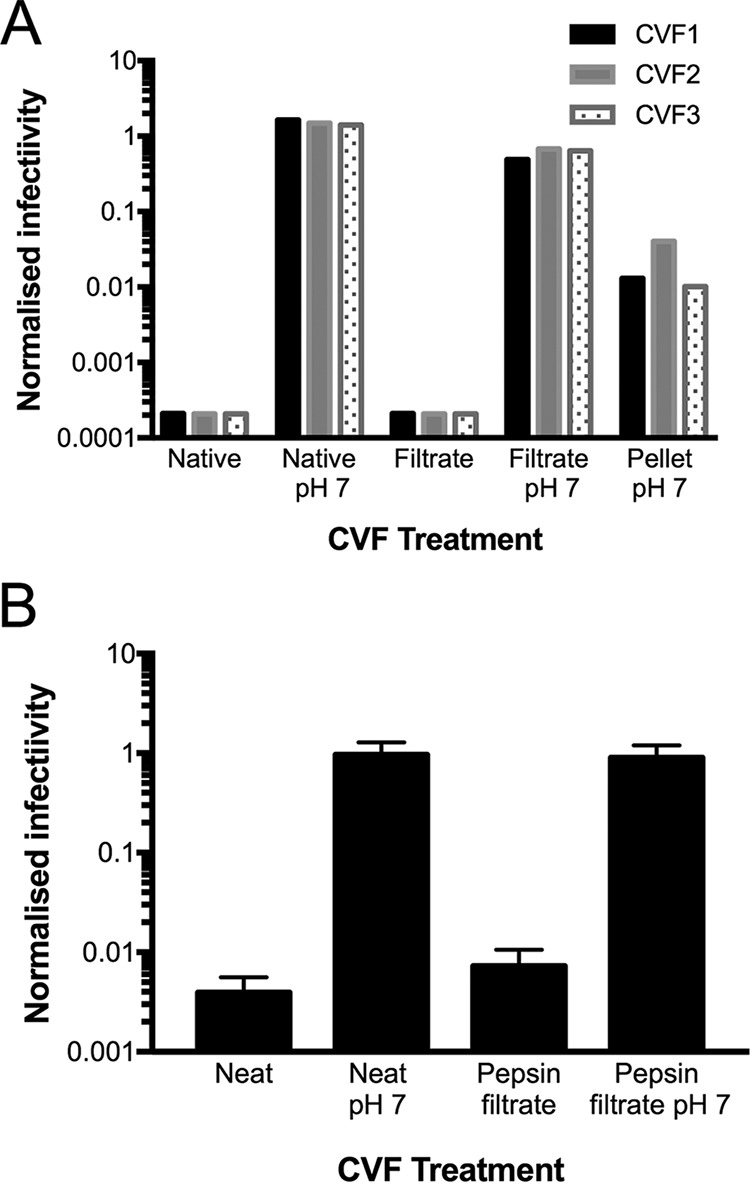
Anti-HIV_Ba-L_ activity was present in the acidic <3-kDa-MW CVF fraction and was retained after digestion with pepsin. (A) Data represent anti-HIV_Ba-L_ activity of pooled native CVF, including unmodified (Native) activity and activity at neutral pH (Native pH 7), separated into a low-molecular-weight <3-kDa fraction by centrifugation through a 3-kDa-MWCO membrane at the original acidic pH (Filtrate) or neutralized pH (Filtrate pH 7) or the ≥3-kDa retentate that was extensively washed with PBS− and resuspended in medium to the original native CVF volume (Pellet pH 7). Data represent results from an individual (*n =* 1) assay of three separate sets of pooled CVF samples. (B) Anti-HIV_Ba-L_ activity of pooled acidic CVF (pH 4.13, clarified by centrifugation, i.e., supernatant), including unmodified (Neat), neutralized pH (Neat pH 7), pepsin-digested, and <3-kDa filtrate recovered by centrifugation and tested at the original acidic pH 4.15 (Pepsin filtrate) or adjusted to neutral pH (Pepsin filtrate pH 7). HIV_Ba-L_ infectivity was normalized to infectivity measured in DMEM-50. The limit of detection was 1.5 × 10^3^ infectious units/ml. Error bars denote standard deviations from *n =* 3 independent assays.

Each of the three sets of pooled native CVF samples inhibited HIV_Ba-L_ infectivity to levels below the detection limit of the assay, representing on average a >7,200-fold decrease in virus infectivity compared to the corresponding neutralized samples (Fig. 4A). After incubation in the acidic <3-kDa fraction, infectious HIV_Ba-L_ was also undetectable, representing on average a >2,900-fold decrease in infectivity compared to the corresponding neutralized filtrate. HIV_Ba-L_ infectivity in neutralized CVF filtrate samples did not dramatically differ from that in native-neutralized CVF (i.e., differed only ~2.5-fold), indicating that most of the HIV_Ba-L_ inhibitory activity in the <3-kDa fraction was present only at low pH. We also examined the ≥3-kDa material retained on the membrane, which was washed and resuspended to the original CVF volume. On average, only 1.4% of the HIV_Ba-L_ inhibitory activity present in native CVF was detected in this fraction in contrast to 40% in the neutralized CVF filtrate (Fig. 4A). These data indicate that the anti-HIV_Ba-L_ activity of native CVF is due to a low-MW factor(s) in the acidic <3-kDa fraction.

To further exclude the possibility that the anti-HIV_Ba-L_ activity in the <3-kDa CVF fraction was due to the presence of low-MW peptides, we next performed HIV-1 inhibition studies with pooled native CVF (final pH of 4.15) that was clarified by centrifugation to obtain “neat” CVF and then subjected to protease digestion followed by separation of the <3-kDa fraction. We used immobilized pepsin to digest proteins present in CVF as it is active at low pH, thus obviating the need to alter sample pH. Protein concentrations were quantified in predigestion and postdigestion CVF samples as well as in postfiltration CVF samples, and the results confirmed removal of ≥3-kDa proteins as well as pepsin-mediated digestion of proteins (see Fig. S1A in the supplemental material). Although there was clear evidence of digestion of peptides compared to the untreated CVF results, peptides that were <3 kDa in molecular weight were not completely removed from CVF (Fig. S1B). We found that the <3-kDa protease-treated neat CVF fraction retained most of its anti-HIV_Ba-L_ activity compared to the original neat CVF and that this inhibitory activity was completely abrogated upon neutralization (Fig. 4B). Taken together with the significant association between HIV-1 inhibitory activity and protonated lactic acid concentrations, these data indicate that protonated lactic acid is a major anti-HIV-1 factor present in native and neat CVF.

10.1128/mSphere.00055-18.1 FIG S1  Monitoring of protein digestion in pooled, clarified cervicovaginal fluid (CVF) (pH 4.2), DMEM containing 10-mg/ml bovine serum albumin (BSA) adjusted to pH 4.7, and media containing 50% FBS (DMEM-50) adjusted to pH 4.5. Samples were digested with pepsin immobilized on beads (pepsin) and then subjected to centrifugation through a 3-kDa-MWCO membrane to collect the <3-kDa sample (Pepsin + Filtrate). Protein concentrations in the “pepsin” and “Pepsin + Filtrate” samples were normalized to the corresponding undigested samples and measured by the (A) Bradford method and (B) bicinchoninic acid assay (BCA). The BCA method but not the Bradford method can detect peptides of <3 kDa. All “Pepsin + Filtrate” samples in panel A had undetectable levels of protein by the Bradford method. Data represent samples from *n =* 1 assay. Download FIG S1, TIF file, 0.1 MB. Copyright © 2018 Tyssen et al. 2018 Tyssen et al. This content is distributed under the terms of the Creative Commons Attribution 4.0 International license.

### Vaginal microbiota, anti-HIV-1 activity, and d+l-protonated lactic acid levels.

**FIG 5:**
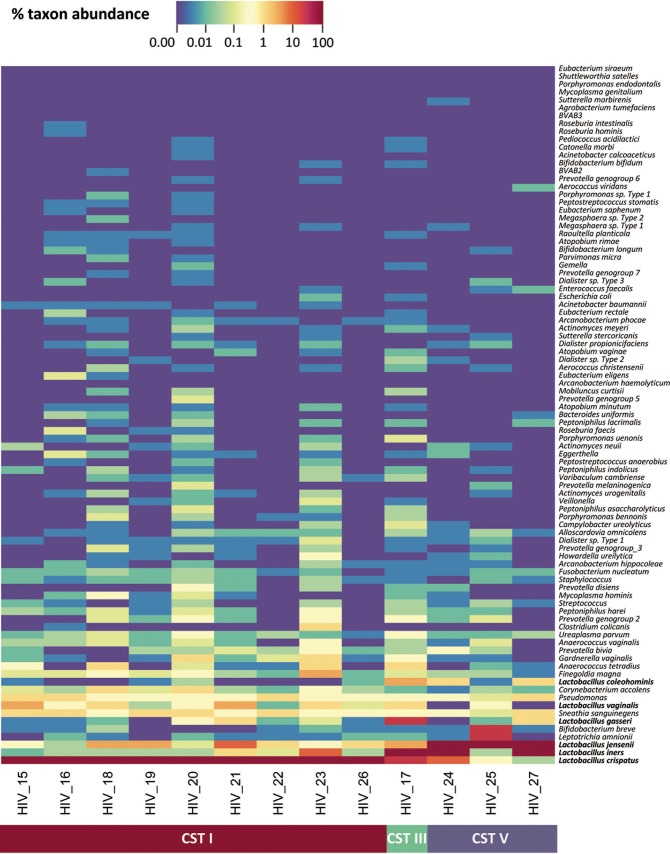
16S rRNA gene sequencing of CVF samples reveals groups with distinct vaginal microbiota, dominated by Lactobacillus crispatus (CST I), L. iners (CST III), or L. jensenii (CST V). Colored bars indicate the abundance of different bacterial species as a proportion of all species in the sample. CVF sample numbers (from *n =* 13 participants) are indicated at the bottom of the heat map.

**FIG 6:**
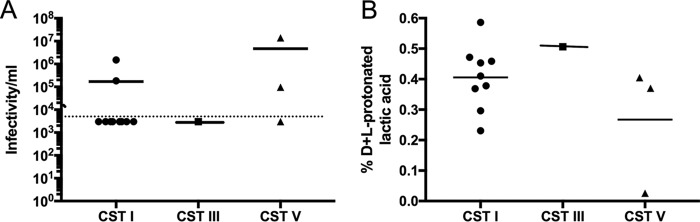
Anti-HIV_Ba-L_ activity and percent d+l-protonated lactic acid (wt/vol) levels of CVF samples with distinct vaginal microbiota dominated by *Lactobacillus* spp. (A) HIV_Ba-L_ infectivity after incubation in CVF samples (HIV_15 to HIV_27) with community state types (CST) dominated by L. crispatus (CST I), L. iners (CST III), or L. jensenii (CST V). Samples represented below the dotted horizontal line were below the detection limit of the infectivity assay. (B) Levels of percent d+l-protonated lactic acid in CVF samples (HIV_15 to HIV_27) categorized as CST (I), CST III, and CST V. Solid horizontal lines represent the means.

For a subset of available CVF samples (*n =* 13), we performed 16S rRNA gene sequencing to determine the relative abundances of vaginal bacteria. To date, five major vaginal bacterial communities (i.e., community state types [CST]) have been identified that are dominated by L. crispatus (CST I), L. gasseri (CST II), L. iners (CST III), or L. jensenii (CST V) or by a diverse set of strict and facultative anaerobic bacterial species with low levels of or no *Lactobacillus* spp. (CST IV-A and IV-B) (37–39). All samples harbored *Lactobacillus* spp. and were categorized as CST I, III, or V, with the majority belonging to CST I (*n =* 9) (Fig. 5). The average percent d+l-lactic acid in CST I samples was 1.14 ± 0.13, and all of these samples had greater levels of d-lactic acid than of l-lactic acid (average d:l ratio of 1.75:1) (Table S2) consistent with previous reports indicating that L. crispatus produces more d-LA (21). CST I samples also showed strong anti-HIV_Ba-L_ activity (Fig. 6A) and high levels of d+l-protonated lactic acid ranging from 0.23% to 0.59% (wt/vol) (Fig. 6B; see also Table S2). Only one sample, HIV_17, was dominated by L. iners (CST III), although that sample contained L. crispatus at a relative abundance of 22.4% (Fig. 5) and had strong anti-HIV_Ba-L_ activity (Fig. 6A), high levels (0.51% [wt/vol]) of d+l-protonated lactic acid (Fig. 6B), and a d:l-lactic acid ratio of 1.75:1 (Table S2). Two of the three CST V samples had strong anti-HIV_Ba-L_ activity and d+l-protonated lactic acid levels that were >0.2% (wt/vol), while the third sample (HIV_25) lacked potent anti-HIV_Ba-L_ activity, contained 10-fold-lower levels of d+l-protonated lactic acid (Fig. 6), and had a pH level of ≥4.5 (Table S2). That sample was classified as CST V; however, Bifidobacterium breve and Leptotrichia amnionii were detected at relative abundances of 25% and 30%, respectively, and a Nugent score of 5 was assigned (Table S2), consistent with a lack of *Lactobacillus* dominance. These data demonstrate that CVF samples with a high relative abundance of *Lactobacillus* spp., particularly L. crispatus, also have high levels of protonated lactic acid and of anti-HIV-1 activity.

## DISCUSSION

Lactic acid is the major organic acid metabolite produced by vaginal *Lactobacillus* spp. and is dramatically depleted in women who are colonized with a diverse set of strict and facultative anaerobes and are at increased risk of acquiring or transmitting HIV (16, 17). In this study, we found a strong and highly significant association between protonated lactic acid levels in native CVF and anti-HIV-1 activity against R5 HIV-1, even when accounting for lactate anion and low pH. Inactivation of HIV-1 was mediated by both l- and d-protonated lactic acid. Anti-HIV-1 activity in acidic CVF could be ascribed to a pH-dependent, protease resistance factor of <3 kDa present in the CVF filtrate, consistent with lactic acid. CVF samples dominated by *Lactobacillus* spp. and displaying the highest anti-HIV-1 activities also had the highest protonated lactic acid levels (>0.2% [wt/vol]). Collectively, these data indicate that protonated lactic acid in CVF is biologically active, is a major HIV-1 virucidal factor present in women with acidic CVF, and may play an important role in modulating HIV transmission or acquisition.

Studies performed by us and others have shown that lactic acid has broad-spectrum *in vitro* HIV virucidal activity (31) and that lactic acid inhibits HIV-1 infection in *ex vivo* tissue models (40). However, those studies did not assess the anti-HIV-1 activity of lactic acid present in native, undiluted CVF samples. Previous studies have reported relatively weak (2-fold to 10-fold) HIV-1 inhibitory activity in cervicovaginal lavage (CVL) fluid (41, 42) or in significantly diluted CVF (36) using the same assay system (HIV_Ba-L_ and TZM-bl cells) compared with the up to 14,000-fold anti-HIV_Ba-L_ activity observed in our study. CVL fluid is significantly diluted vaginal fluid and is usually recovered using 5 to 10 ml of normal saline solution at neutral pH (a 5-fold to 10-fold dilution, assuming 1 to 2 ml of native CVF in the vagina) and further diluted by at least 1:4 in medium prior to addition to HIV target cells (41, 42). Similarly, in another study that used CVF (36), the vaginal fluid was diluted by at least 20-fold in culture medium before use. Experiments using CVL or significantly diluted CVF in buffered medium are highly unlikely to detect the anti-HIV-1 activity of protonated lactic acid because of dilution well below the 0.3% (wt/vol) lactic acid (or 0.14% protonated lactic acid) threshold for HIV-1 virucidal activity (31) and because protonated lactic acid levels would be further decreased due to neutralization of the original sample acidity (31). In contrast, we incubated high-titer HIV-1 in native CVF, minimally diluted by the addition of virus, which allowed us to maximize detection of HIV-1 inhibitory activity in the native fluid.

Our analysis shows a significant association of protonated lactic acid levels with anti-HIV_Ba-L_ activity in CVF that is independent of the lactate anion and pH and that lactate anion and pH alone are not significantly associated with anti-HIV activity. The lack of association with lactate anion was expected given that this form of lactic acid is devoid of anti-HIV-1 activity (31). In contrast, the lack of association of anti-HIV activity in CVF with pH is surprising given that protonated lactic acid levels dominate at pH below the pK_a_ of lactic acid and that protonated lactic acid is absolutely required for HIV-1 inactivation (31). It is likely that a significant association between pH and anti-HIV_Ba-L_ could not be discerned in our study given the weak anti-HIV-1 activity of low pH and/or the narrow range of pH values in the evaluated samples that limited the power to detect an association in our data set. In support of the former possibility, our previous study demonstrated that under identical assay conditions, the presence of protonated lactic acid results in a 10^5^-fold drop in HIV_Ba-L_ infectivity compared to a 29-fold decrease for media acidified to the same pH with HCl (31).

We have previously demonstrated and discussed in detail the implausibility of either H_2_O_2_ or acetic acid playing a significant antimicrobial role in the vagina (19, 30, 43, 44). While protonated lactic acid inactivates HIV-1 directly (31), the precise mechanism of action is unknown, although inactivation occurs in the absence of virion disruption and loss of gp120 (45). Aside from protonated lactic acid, other possible antiviral factors in CVF are AMPs (35, 36). The main AMPs reported in the lower female reproductive tract include α-defensins (human neutrophil peptide 1 [HNP1] to HNP4 and human defensin 5 [HD5] and HD6), human β-defensin 1 (HBD1) to HBD5, elafin, secretory leukocyte protease inhibitor (SLPI), cathelicidin (LL37), calprotectin, cathepsin G, lysozyme, and lactoferrin (35, 36, 46, 47). However, the role of AMPs in HIV-1 inactivation is controversial (recently reviewed by Cole and Cole) (47). While AMPs can block virus entry (e.g., by downmodulating chemokine receptors on target cells as reported for LL-37) (47) or directly target the HIV particle *in vitro* (e.g., by lactoferrin and elafin) (36, 48), they can also enhance HIV-1 infection *in vivo* through their immune modulatory effects (47). Further, AMP concentrations shown to elicit anti-HIV-1 effects *in vitro* (usually in microgram-per-milliliter quantities) may not represent the lower physiological levels present in vaginal fluid, as reported for HNPs, HBD-2, and lysozyme (35, 47), and may actually be cytotoxic to target cells rather than specifically inhibiting HIV-1 (e.g., HNPs) (47). In contrast, the *in vitro* anti-HIV activity of LL37 and elafin is at physiological levels (47). However, while many AMPs are not individually present at anti-HIV levels in CVF, they can act in concert to inhibit HIV-1 *in vitro* (36). Regardless, all of the AMPs mentioned above have molecular weights that are >3 kDa (36, 46, 47) and would not be expected to be present in the <3-kDa filtrate. Indeed, we were able to demonstrate that most of the anti-HIV-1 activity was retained in the acidic <3-kDa filtrate of CVF and persisted even after pepsin digestion, suggesting that HIV-1 inactivation by CVF is unlikely to be mediated by known AMPs. However, the CVF peptides of <3 kDa in the filtrate were not completely removed in our experiments (see Fig. S1 in the supplemental material). Accordingly, we cannot exclude the possibility that uncharacterized peptides of <3 kDa, including those derived from AMP proteolysis, contribute to the anti-HIV-1 observed in our study, particularly in minimally diluted CVF samples. Finally, apart from AMPs, purified exosomes from vaginal fluid have been reported to inhibit an early stage of the HIV life cycle (49); however, the activity of exosomes is likely to be distinct from the pH-dependent anti-HIV-1 activity reported here in native as well as neat CVF. Furthermore, exosomes would be absent from the <3-kDa filtrate.

While our data indicate that lactobacillus-produced protonated lactic acid is a major mediator of anti-HIV-1 activity in native CVF, it is possible that different *Lactobacillus* spp. may confer different levels of protection against HIV. Women with L. crispatus-dominated vaginal microbiota have a lower risk of acquiring HIV from their male partners (4, 5) than women colonized with L. iners. While the participants in this study were not selected based on race, most of the women identified as non-Hispanic white or Asian and all of the CVF samples subjected to 16S rRNA gene sequencing were dominated by *Lactobacillus* spp., the majority harboring L. crispatus. The high prevalence of *Lactobacillus* spp. is similar to that seen in our previous study performed using CVF samples from Johns Hopkins University female participants of reproductive age and is consistent with the low BV prevalence in this study population (22). Larger studies of asymptomatic women in distinct geographical regions would be of interest to determine whether protonated lactic acid has an anti-HIV role in CVF from women in various countries.

CVF dominated by L. crispatus and L. gasseri produced both d- and l-lactic acid as previously described for axenic *Lactobacillus* cultures (21). Axenic cultures of L. iners produce only l-lactic acid (21). The sole L. iners-dominated CVF sample (HIV_17) contained both lactic acid isomers, although the d:l-lactic acid ratio (0.79:1) was lower than in CST I and CST V samples, and the 22.4% relative abundance of L. crispatus (Fig. 5A) may explain the presence of d-lactic acid in the sample (see Table S2 in the supplemental material). The majority of characterized microbiota CVF samples had potent anti-HIV-1 activity except for HIV_25 (Fig. 1), harboring L. jensenii, which also had lowest levels of d+l-protonated lactic acid (Fig. 5; see also Table S2). The presence of significant (>50%) proportions of other bacteria, namely, Bifidobacterium breve and Leptotrichia amnionii, may account for the low protonated lactic acid level and low anti-HIV-1 activity in this sample. The important anti-HIV-1 role of d+l-protonated lactic acid is highlighted by the results from sample HIV_12. Unfortunately, the microbiota of this sample was not characterized by 16S rRNA gene sequencing. However, while HIV_12 had a Nugent score of 2 and a d+l-lactic acid level of 1.1% (wt/vol) (Tables S1 and S2), which are characteristics consistent with *Lactobacillus*-dominated microbiota (22), this sample lacked anti-HIV-1 activity (Fig. 1). Notably, the pH of this sample was high (pH 5.43) and it had the second-lowest d+l-protonated lactic acid level (only 0.03% [wt/vol]) of all samples analyzed (Table S2). A limitation of our study was that since most of the samples for which the microbiota was characterized belonged to CST I, we were unable to determine whether there was a significant difference in the presence of protonated lactic acid and thus anti-HIV-1 activity in CVF samples dominated with L. crispatus compared to less-protective *Lactobacillus* spp. in other CSTs. Future studies in populations with *Lactobacillus*-dominated vaginal microbiota may be able to address this issue.

Our findings of a strong association between HIV-1 inactivation by CVF and protonated lactic acid levels suggest that protonated lactic acid, through its direct anti-HIV-1 activity, may be responsible in part for reducing the vaginal HIV load observed in HIV-positive women colonized with *Lactobacillus*-dominated vaginal microbiota compared to women lacking significant numbers of *Lactobacillus* spp., such as in cases of BV (5–11). A reduction of the levels of infectious HIV particles in the vaginal lumen would be expected to lower the risk of transmission from HIV-positive women to their male partners or to neonates during birth. In male-to-female transmission, HIV is deposited by semen, which transiently neutralizes the vagina (43, 50). Seminal plasma, a surrogate for semen, attenuates the HIV virucidal activity of protonated lactic acid *in vitro*, likely due to its strong buffering capacity that increases pH and decreases protonated lactic acid (31). Thus, whether protonated lactic acid in CVF is able to block HIV male-to-female transmission depends on whether lactobacilli can maintain vaginal acidity very near the vaginal epithelial surface and, if acidity is lost, how rapidly *Lactobacillus* spp. can reacidify at this site before HIV can reach and infect HIV target cells (43). The postcoital pH gradient relative to the epithelial surface is unknown. The rate of acidification by vaginal *Lactobacillus* spp. is ~0.5 pH units/h and is consistent with reacidification of the vagina within several hours following coital deposition of semen (51). The time required for HIV to enter and infect target cells in vagina colonized with *Lactobacillus* spp. is unknown, although vaginal infection of rhesus macaques with high-titer cell-free simian immunodeficiency virus can be demonstrated within 60 min of exposure (52). However, macaques rarely have acidic vaginas and do not harbor *Lactobacillus* spp., and their vaginal pH is similar to that in women with BV (53, 54). Additionally, native CVF traps HIV-1 in a pH- and d-lactic acid concentration-dependent manner, which could further reduce the ability of the virus to enter and infect cells in the vaginal mucosa, especially in women with a high d:l-lactic acid ratio indicative of L. crispatus-dominated microbiota (55, 56).

We examined the ability of CVF to inactivate cell-free HIV-1; however, cell-associated HIV is also present in the cervicovaginal and seminal fluids of infected individuals that could act as a vehicle to transmit HIV (50). HIV target cells (i.e., human lymphocytes, monocytes, and macrophages) are immobilized at between pH 5.5 to 6.0 and are killed when the pH decreases to levels below 5.5 (57). Application of a microbicide, acidified to pH 3.9 and designed to maintain vaginal acidity in the presence of semen, significantly reduced vaginal transmission of cell-associated HIV-1 in the HuPBL-SCID mouse model (57). Those experiments were performed with an acidifying microbicide that maintained a low pH but lacked lactic acid (57) and that was thus orders of magnitude less potent in inactivating cell-free HIV (31). Lactic acid also kills cells, with >0.1% lactic acid at low pH reported to reduce cell viability by >90% (17). Thus, it is likely that women with *Lactobacillus*-dominated vaginal microbiota and high levels of protonated lactic acid could potentially inactivate both cell-free and cell-associated HIV.

We have shown that protonated lactic acid in acidic, native CVF mediates potent anti-HIV-1 activity. Protonated lactic acid may confer additional antimicrobial and immune modulatory effects in the vagina, previously described in *in vitro* and *ex vivo* studies (17, 26–30, 40). For example, protonated lactic acid mediates direct immune modulatory effects on cervicovaginal epithelial cells that could dampen the genital inflammation that promotes HIV acquisition (17) and has bactericidal activity against 17 different BV-associated bacterial species without affecting vaginal *Lactobacillus* spp. (30). The results of this study may have broader significance for the design of protonated-lactic-acid-based microbicides or *Lactobacillus*-based probiotics as adjuncts to target HIV infections and other STIs (26–29) as well as for strategies to reduce the prevalence of BV and low-*Lactobacillus*-species CSTs in women and thereby may indirectly reduce their risk of acquiring or transmitting HIV (8, 13–15, 30). In addition, our study data suggest that changing modifiable behaviors and practices that increase HIV risk in women, including intravaginal cleaning with soap, which elevates the vaginal pH and disrupts the optimal lactobacillus-dominated vaginal microbiota (58), may help combat HIV by promoting a high-lactic-acid environment.

## MATERIALS AND METHODS

### Collection of human CVF.

CVF collection was performed as published previously (33, 45, 59, 60). Briefly, undiluted native CVF was obtained from women who were of reproductive age (18 to 45 years old) and in good general health by using a self-sampling menstrual collection device (Instead Softcup). Ethical approval was obtained from the Homewood Institutional Review Board, Johns Hopkins University (JHU) (approval HIRB00000526), and from the Alfred Ethics Committee (Project 80/13). Informed consent of participants was obtained after the nature and possible consequences of the study were explained. Participants inserted the device into the vagina for at least 30 s, removed it, and placed it into a 50-ml centrifuge tube. Samples were centrifuged at 200 × *g* for 1 min to collect the secretions. Samples were collected at random times throughout the menstrual cycle, and cycle phase was estimated based on the last menstrual period date normalized to a 28-day cycle. No samples were ovulatory (based on visual observation [i.e., none exhibited spinnbarkeit]), and no samples were bloody or nonuniform in color or consistency. Donors stated that their last menstrual period had ended at least 3 days prior to donating and that they had not used vaginal products or participated in intercourse within 24 h prior to donating. Donor demographics are reported in Table S1 in the supplemental material.

### Preparation of CVF aliquots.

Native CVF samples were kept on ice immediately after sample collection until all aliquots were prepared. To provide better sample uniformity across conditions, each sample was first stirred gently with a pipette tip for 20 s, and aliquots were prepared in 5-µl-to-10-µl increments using a capillary-tube-positive displacement micropipette (Wiretrol; Drummond Scientific, Broomall, PA). Native CVF, prepared as 50-µl aliquots, was used to test the following conditions: (i) native (unmodified) CVF, (ii) neutralized CVF, (iii) 3-fold-diluted CVF (for a subset of samples), (iv) 9-fold-diluted CVF, and (v) 27-fold-diluted CVF. Precise dilution factors for these samples were calculated based on sample weight before and after dilution, taking into account the addition of the viral inoculum (5% volume). For condition ii, CVF was neutralized (average pH, 7.3 ± 0.2; range, 6.9 to 7.6) by incremental addition of 5 N NaOH (up to 3% volume) and pH was measured using a micro-pH electrode (Microelectrodes, Inc., Bedford, NH). For conditions iii, iv, and v, CVF was diluted approximately 3-, 9-, and 27-fold, respectively, with unbuffered 0.9% saline solution. Pilot studies demonstrated that this dilution range is sufficient to produce only partial HIV-1 inactivation in most CVF samples. Samples were placed in sterile, conical-bottom polypropylene tubes (Sarstedt 72.693.105; Sarstedt, Newton, NC) and frozen at −80°C. A second set of 25-µl aliquots was similarly prepared for pH measurements after addition of virus stock (5% volume). The pH of all CVF samples was measured in air (i.e., under aerobic and hypocapnic conditions). Accordingly, the pH values were expected to be marginally higher (by approximately 0.3 pH units) than the pH values measured *in situ* (22).

### Biochemical characterization of CVF samples.

Aliquots of CVF were prepared for biochemical characterization by diluting native CVF samples 50-fold with unbuffered 0.9% saline solution, gentle vortex mixing, and centrifuging at 1,000 × *g* for 2 min to collect “diluted CVF cell-free supernatant,” which was then stored at −80°C. Prior to use, aliquots were thawed, and serial dilutions were prepared with unbuffered 0.9% saline solution within the linear range of each assay. Lactic acid levels were measured using a d-lactic acid/l-lactic acid kit (R-Biopharm, Darmstadt, Germany) following the manufacturer’s protocol adapted to a 96-well format. This method measures the concentration of lactate anion after neutralization of the sample to pH 7 to 9 to calculate the total concentration of lactic acid in the sample. At pH levels of <7, samples contain the lactate anion and protonated lactic acid, the sum of which we refer to as lactic acid. The protonated form of lactic acid present in CVF samples was calculated from the sample pH and lactic acid concentration using the Henderson-Hasselbalch equation as previously described (22, 35). For these calculations, we made the assumption that the pK_a_ value for lactic acid in CVF is 3.86. The biochemical characteristics of CVF samples are summarized in Table S2.

### Cells.

TZM-bl and 293T cells were obtained through the NIH AIDS Research and Reference Reagent Program and were cultured in DMEM-10 (DMEM containing 10% fetal bovine serum [FBS]) as previously described (31). Peripheral blood mononuclear cells (PBMCs) were purified from blood bank packs by Ficoll-Paque density gradient centrifugation from the blood of HIV-seronegative donors obtained from the Australian Red Cross Blood Service (Melbourne, Australia) as previously published (61). Purified PBMCs were stimulated for 2 to 4 days with 10 µg/ml phytohemagglutinin (PHA) in RF-10 medium (31) supplemented with 10 U/ml recombinant human interleukin-2 (hIL-2 [Roche]; Sigma-Aldrich, Castle Hill, NSW, Australia). All cells were maintained at 37°C in 5% CO_2_.

### Virus strains.

HIV_Ba-L_, a CCR5 (R5)-using subtype B laboratory strain of HIV-1 (62), and pRHPA.c/2635, a molecular clone from a subtype B, R5 transmitted/founder strain from a female subject which had been acquired heterosexually (34), were obtained through the NIH AIDS Research and Reference Reagent Program. HIV_RHPA_ was generated from pRHPA.c/2635 by calcium phosphate transfection of 293T cells (63). HIV_Ba-L_ and HIV_RHPA_ were propagated in PHA-stimulated PBMCs as previously published (31) with the following modifications. At day 3 or 4 postinfection, the supernatant was removed from the HIV-1-infected PBMCs by centrifugation at 600 × *g* for 10 min, and PBMCs were replenished with 10 ml of Opti-MEM (Gibco, Thermo Fisher Scientific, Scoresby, Victoria, Australia) in the absence of FBS and incubated for a further 3 days at 37°C in 5% CO_2_. At day 7 postinfection, virus supernatant was clarified by centrifugation at 2,100 × *g* for 10 min and concentrated 200-fold using a Vivaspin-20 concentrator (Sartorius Stedim, Dandenong South, Victoria, Australia) according to the manufacturer’s instructions. FBS was then added to the viral supernatant to reach a final concentration of 10%, and aliquots were stored at −80°C. HIV_Ba-L_ and HIV_RHPA_ stock titers were determined in TZM-bl cells by counting β-galactosidase-producing cells as described below.

### HIV-1 inhibition assays.

The HIV-1 inhibitory activity of CVF samples was assessed using an infectivity assay. CVF samples were thawed and maintained at 4°C before each sample was spiked with a virus inoculum equal to 5% of the CVF volume where the titer was 2.0 × 10^7^ infectious units for HIV_Ba-L_ and 1.0 × 10^7^ infectious units for HIV_RHPA_. Spiked CVF samples were thoroughly mixed for 30 s by the use of a pipette tip and incubated for 10 min on a 37°C heat block. In parallel, separate wells containing DMEM-50 (DMEM containing 50% FBS) were included in each assay as a control to determine the virus titer in the absence of CVF. Following incubation, samples were neutralized by 20-fold dilution in cold DMEM-DEAE (DMEM-10 supplemented with 25 mM HEPES buffer [Gibco], 10 µg/ml ciprofloxacin, and 20 µg/ml DEAE-dextran [GE Healthcare, Silverwater, NSW, Australia]). Since we have previously shown that HIV-1 inactivation by protonated lactic acid is both rapid and irreversible (31), this allowed detection of virucidal activity in the sample. For logistical reasons, diluted samples were divided into aliquots and stored at −80°C for a maximum of 4 h prior to quantification of infectious HIV-1 using TZM-bl indicator cells. Our pilot studies confirmed that viral titers in samples subjected to short-term storage at −80°C (and one freeze-thaw cycle) for up to a day were comparable to those of samples stored at 4°C. Each neutralized sample was tested for infectious virus quantified by β-galactosidase staining of TZM-bl cells (64). Six 5-fold dilutions of each neutralized sample were performed in DMEM-DEAE, and the diluted samples were added in triplicate to wells of 96-well tissue culture plates (Nunclon, Denmark) that were seeded 24 h prior to infection with 2.0 × 10^4^ cells per well. Following a 2-day incubation at 37°C in 5% CO_2_, TZM-bl cells were washed twice in Dulbecco’s phosphate-buffered saline without calcium and magnesium (PBS−), treated with fixing solution (0.2% glutaraldehyde, 1% formaldehyde, PBS−) for 5 min at room temperature, and then incubated for 2 h at 37°C in staining solution (400 µg/ml of 5-bromo-4-chloro-3-indolyl-beta-d-galactopyranoside, 2 mM MgCl_2_, 4 mM potassium ferrocyanide, 4 mM potassium ferricyanide, PBS−). Mock-infected cells, treated identically to test samples but lacking virus, were included and used to determine the background. HIV-1 titers were determined by counting blue-colored cells (in the range of 0 to 200 blue-forming cells/well and without evidence of cytotoxicity) as visualized by light microscopy. To accurately quantify levels of infectious HIV-1 present in samples and to minimize potential confounding effects due to factors, including AMPs, that can either enhance or diminish HIV levels (36, 47), we calculated the HIV-1 titer from two consecutive dilutions with titers that did not differ more than 25%. The interassay coefficient of variation (CV) of our HIV-1 inhibition assay, as determined for HIV_Ba-L_ infectivity in DMEM-50, is 17%. This value is in a range similar to that previously reported for HIV-1 assays in TZM-bl cells (65).

### Preparation of CVF filtrate and retentate.

Three sets of pooled CVF samples were prepared from donor samples that individually exhibited low pH. Lactic acid concentrations of samples were quantified as described above. Aliquots of native and neutralized pooled CVF, prepared as described above, were set aside. The remaining volumes of the native, pooled CVF samples were filtered through Amicon Ultra 0.5 centrifugal filters (Sigma, St. Louis, MO) with a 3-kDa MWCO at 21,000 × *g* for 30 min. The resulting filtrate was divided into two fractions, one of which was neutralized as performed for native CVF. The retentate was washed twice with PBS− and resuspended to the original volume of the prefiltered CVF. All aliquots were stored at −80°C until use in HIV-1 inhibition studies.

### Preparation of pepsin-treated CVF.

Pooled native CVF was prepared as described above and clarified by centrifugation at 20,800 × *g* for 30 min at 4°C to recover “neat” CVF supernatant. The pH of the pooled neat CVF was measured before the CVF was subjected to digestion using pepsin immobilized on cross-linked agarose beads (Thermo Fisher Scientific). Pepsin beads were prepared by centrifugation performed three times at 1,000 × *g* for 3 min in 20 mM sodium acetate at pH 4.2 to remove the preservative before beads were resuspended directly in pooled neat CVF supernatant at a volume ratio of 1:4 pepsin/CVF. Pepsin digestion of neat CVF supernatant was performed for 24 h at 37°C with constant gentle mixing.

Immobilized pepsin was removed by centrifugation at 1,500 × *g* for 4 min at room temperature, and the recovered CVF supernatant was filtered through an Amicon Ultra-0.5 Ultracel-3 membrane 3-kDa concentrator (Merck Millipore, Bayswater, Victoria, Australia) at 15,000 × *g* for 1 h at 4°C. Bovine serum albumin (BSA) Fraction V (Sigma-Aldrich, Castle Hill, NSW, Australia), prepared at 10 mg/ml in sterile water, and DMEM-50 were included as controls. Pre- and post-pepsin digestion and post-3-kDa-filtration samples were subjected to HIV-1 infectivity analyses, as described above, and protein concentration determined using Bio-Rad Protein Assay reagent (Bio-Rad, Gladesville, NSW, Australia) and the Micro BCA Protein Assay (Thermo Fisher Scientific).

### Nugent scoring and microbiota analysis.

A vaginal smear slide was prepared for Gram staining and Nugent scoring. Gram-stained slides were viewed under a 100× objective, and Nugent scores were calculated as described previously (66). For microbiota characterization, 10 µl of native CVF was added to 90 µl of transfer media (eSwab; BD, Franklin Lakes, NJ) and stored at −80°C. DNA extractions were performed using a PowerMag microbiome RNA/DNA isolation kit (Mo Bio Laboratories, Inc., Carlsbad, CA), and the reaction mixtures were processed using a Hamilton Microlab Star robotic platform (Hamilton, Reno, NV). Two controls, comprising a mixture of 24 stool samples and a mixture of 24 vaginal samples, were included on all sequencing plates with test samples. The taxonomy and community composition of these controls were compared to data generated from the same controls on all previous runs and used as a pass/fail in the quality control process. Briefly, CVF samples were pelleted by centrifugation for 1 min at 4,500 × *g* and the pellet was mixed with 650 µl of PowerMag lysis solution containing 2.4% (vol/vol) β-mercaptoethanol. Bacteria were disrupted using a Qiagen TissueLyser and a 96-well glass bead plate at 30 Hz for 20 min followed by centrifugation at 4,500 × *g* for 6 min and transfer of the clarified supernatant to a clean plate. PowerMag Inhibitor Removal Solution (150 µl) was then added to the clarified supernatants, and the reaction mixtures were incubated at 4°C for 5 min. The plate was centrifuged at 4,500 × *g* for 6 min, and then 850 µl of each sample was transferred to a fresh 2-ml 96-well plate. A 850-µl volume of PowerMag Binding Solution and 20 µl of PowerMag Magnetic Bead Solution were added to each sample, and the mixture was homogenized by gentle vortex mixing for 10 min. Samples were washed three times by placing the plate on a magnetic plate for 5 min to collect DNA-bound beads at the bottom of the wells and by replacing the supernatant with 500 µl of ClearMag wash buffer for each wash. Beads were then resuspended in 50 µl of ClearMag RNase-free water and heated to 65°C for 5 min to separate DNA from the beads. Beads were collected a second time on the magnetic plate, and the samples containing microbial DNA were transferred to a PowerMag microplate. The vaginal microbiota composition and structure were characterized as previously published (67) by amplification of the V3-V4 regions of the 16S rRNA gene and by sequencing of the bar-coded amplicons on an Illumina MiSeq instrument using the 300-bp paired-end read protocol as recommended by the manufacturer (Illumina, San Diego, CA). Sequence analysis and taxonomic assignments were performed using a custom pipeline freely available on GitHub (https://github.com/cwzkevin/MiSeq16S). CST assignments were performed as previously reported (37).

### Statistical modeling of associations between HIV-1 infectivity and protonated lactic acid and lactate concentrations and pH.

Generalized linear mixed modeling (GLMM) was used to estimate the association between HIV-1 infectivity and CVF sample lactic acid concentration, pH, and neutralization. Infectivity data below the limit of detection (LOD), defined as 3 times the background (i.e., 6 blue forming cells), were assigned a value of LOD/2 (68). Given the discrete (nonnormal) nature of the outcome data in this instance (i.e., HIV-1 infectivity counts per milliliter), GLMM was generalized by a Poisson distribution and log-link function. To account for the inherent dependency in the data from repeated measurements of HIV-1 infectivity after subsequent CVF sample treatment, GLMM analyses specified a participant-specific random intercept—effectively modeling the data corresponding to the heterogeneity between participants in the person-specific HIV-1 infectivity levels which induce correlation across serial measurements. The multilevel structures of the GLMM data were such that study participants represented level-2 units and their serial HIV-1 infectivity measurements level-1 observations. A gamma distribution was assumed for the random intercept in all GLMM analyses. Given the restrictive "equidispersion" assumption of the Poisson distribution, normal-based bootstrapped standard errors (69) (*n =* 1,000 replications) were estimated in GLMMs to provide more-conservative inference data that were less vulnerable to overdispersion. Where bootstrapped standard errors could not be estimated, robust standard errors were used (70). In terms of the fixed part of GLMM analyses, the functional form of each association was explored for protonated lactic acid, lactate anion, and pH, with either linear or polynomial (quadratic) effects modeled depending on model fit. Wald chi-square statistics were used to provide inference in assessing the functional form of associations (likelihood ratio tests could not be estimated given the nonparametric approach to inference used [bootstrapping]). Model-based postestimation predicted HIV-1 infectivity counts were produced and plotted for each respective unadjusted model; predicted HIV-1 infectivity counts were based on the fixed part of the GLMMs and assumed a mean level of HIV-1 infectivity for the random intercept. Given the number of derivations and subsequent estimations of effect undertaken with respect to protonated lactic acid and lactate anion, statistical significance was determined conservatively at the 1% level. Stata statistical software package 2013, version 14.2 (StataCorp LP, College Station, TX, USA), was used in all statistical modeling analyses, and GraphPad PRISM 7 (La Jolla, CA) was used to produce plots and graphs.

### Accession number(s).

Data from the study have been deposited in the NCBI database (BioProject identifier [ID] PRJNA430827) (SRP132050). Vagina microbiota sequence read archive (SRA) data for samples HIV_15 to HIV_27 have been deposited under accession numbers SAMN08428826, SAMN08428827, SAMN08428828, SAMN08428829, SAMN08428830, SAMN08428831, SAMN08428832, SAMN08428833, SAMN08428834, SAMN08428835, SAMN08428836, SAMN08428837, and SAMN08428838.

## References

[1] Hladik F, Hope TJ 2009 HIV infection of the genital mucosa in women. Curr HIV AIDS Rep 6:20–28. doi:10.1007/s11904-009-0004-1. 19149993

[2] Anderson D, Politch JA, Pudney J 2011 HIV infection and immune defense of the penis. Am J Reprod Immunol 65:220–229. doi:10.1111/j.1600-0897.2010.00941.x. 21214659 PMC3076079

[3] Buve A, Jespers V, Crucitti T, Fichorova RN 2014 The vaginal microbiota and susceptibility to HIV. AIDS 28:2333–2344. doi:10.1097/QAD.0000000000000432. 25389548

[4] Gosmann C, Anahtar MN, Handley SA, Farcasanu M, Abu-Ali G, Bowman BA, Padavattan N, Desai C, Droit L, Moodley A, Dong M, Chen Y, Ismail N, Ndung'u T, Ghebremichael MS, Wesemann DR, Mitchell C, Dong KL, Huttenhower C, Walker BD, Virgin HW, Kwon DS 2017 Lactobacillus-deficient cervicovaginal bacterial communities are associated with increased HIV acquisition in young South African women. Immunity 46:29–37. doi:10.1016/j.immuni.2016.12.013. 28087240 PMC5270628

[5] Borgdorff H, Tsivtsivadze E, Verhelst R, Marzorati M, Jurriaans S, Ndayisaba GF, Schuren FH, van de Wijgert JH 2014 Lactobacillus-dominated cervicovaginal microbiota associated with reduced HIV/STI prevalence and genital HIV viral load in African women. ISME J 8:1781–1793. doi:10.1038/ismej.2014.26. 24599071 PMC4139719

[6] Sha BE, Zariffard MR, Wang QJ, Chen HY, Bremer J, Cohen MH, Spear GT 2005 Female genital-tract HIV load correlates inversely with Lactobacillus species but positively with bacterial vaginosis and Mycoplasma hominis. J Infect Dis 191:25–32. doi:10.1086/426394. 15592999

[7] Mitchell C, Balkus JE, Fredricks D, Liu C, McKernan-Mullin J, Frenkel LM, Mwachari C, Luque A, Cohn SE, Cohen CR, Coombs R, Hitti J 2013 Interaction between lactobacilli, bacterial vaginosis-associated bacteria, and HIV type 1 RNA and DNA genital shedding in U.S. and Kenyan women. AIDS Res Hum Retroviruses 29:13–19. doi:10.1089/AID.2012.0187. 23020644 PMC3537306

[8] Frank DN, Manigart O, Leroy V, Meda N, Valéa D, Zhang W, Dabis F, Pace NR, Van de Perre P, Janoff EN 2012 Altered vaginal microbiota are associated with perinatal mother-to-child transmission of HIV in African women from Burkina Faso. J Acquir Immune Defic Syndr 60:299–306. doi:10.1097/QAI.0b013e31824e4bdb. 22343176 PMC6384121

[9] Coleman JS, Hitti J, Bukusi EA, Mwachari C, Muliro A, Nguti R, Gausman R, Jensen S, Patton D, Lockhart D, Coombs R, Cohen CR 2007 Infectious correlates of HIV-1 shedding in the female upper and lower genital tracts. AIDS 21:755–759. doi:10.1097/QAD.0b013e328012b838. 17413697

[10] Mane A, Angadi M, Vidhate P, Bembalkar S, Khan I, Bichare S, Ghate M, Thakar M 2017 Characterization of vaginal lactobacilli from HIV-negative and HIV-positive Indian women and their association with genital HIV-1 shedding. J Med Microbiol 66:1471–1475. doi:10.1099/jmm.0.000599. 28945188

[11] Kindinger LM, Bennett PR, Lee YS, Marchesi JR, Smith A, Cacciatore S, Holmes E, Nicholson JK, Teoh TG, MacIntyre DA 2017 The interaction between vaginal microbiota, cervical length, and vaginal progesterone treatment for preterm birth risk. Microbiome 5:6. doi:10.1186/s40168-016-0223-9. 28103952 PMC5244550

[12] Spiegel CA, Amsel R, Holmes KK 1983 Diagnosis of bacterial vaginosis by direct gram stain of vaginal fluid. J Clin Microbiol 18:170–177. 6193137 10.1128/jcm.18.1.170-177.1983 PMC270763

[13] Taha TE, Hoover DR, Dallabetta GA, Kumwenda NI, Mtimavalye LA, Yang LP, Liomba GN, Broadhead RL, Chiphangwi JD, Miotti PG 1998 Bacterial vaginosis and disturbances of vaginal flora: association with increased acquisition of HIV. AIDS 12:1699–1706. doi:10.1097/00002030-199813000-00019. 9764791

[14] Atashili J, Poole C, Ndumbe PM, Adimora AA, Smith JS 2008 Bacterial vaginosis and HIV acquisition: a meta-analysis of published studies. AIDS 22:1493–1501. doi:10.1097/QAD.0b013e3283021a37. 18614873 PMC2788489

[15] Cohen CR, Lingappa JR, Baeten JM, Ngayo MO, Spiegel CA, Hong T, Donnell D, Celum C, Kapiga S, Delany S, Bukusi EA 2012 Bacterial vaginosis associated with increased risk of female-to-male HIV-1 transmission: a prospective cohort analysis among African couples. PLoS Med 9:e1001251. doi:10.1371/journal.pmed.1001251. 22745608 PMC3383741

[16] Aldunate M, Srbinovski D, Hearps AC, Latham CF, Ramsland PA, Gugasyan R, Cone RA, Tachedjian G 2015 Antimicrobial and immune modulatory effects of lactic acid and short chain fatty acids produced by vaginal microbiota associated with eubiosis and bacterial vaginosis. Front Physiol 6:164. doi:10.3389/fphys.2015.00164. 26082720 PMC4451362

[17] Hearps AC, Tyssen D, Srbinovski D, Bayigga L, Diaz DJD, Aldunate M, Cone RA, Gugasyan R, Anderson DJ, Tachedjian G 2017 Vaginal lactic acid elicits an anti-inflammatory response from human cervicovaginal epithelial cells and inhibits production of pro-inflammatory mediators associated with HIV acquisition. Mucosal Immunol 10:1480–1490. doi:10.1038/mi.2017.27. 28401934

[18] Aroutcheva A, Gariti D, Simon M, Shott S, Faro J, Simoes JA, Gurguis A, Faro S 2001 Defense factors of vaginal lactobacilli. Am J Obstet Gynecol 185:375–379. doi:10.1067/mob.2001.115867. 11518895

[19] Tachedjian G, O’Hanlon DE, Ravel J 2018 The implausible “in vivo” role of hydrogen peroxide as an antimicrobial factor produced by vaginal microbiota. Microbiome 6:29. doi:10.1186/s40168-018-0418-3. 29409534 PMC5801833

[20] Boskey ER, Cone RA, Whaley KJ, Moench TR 2001 Origins of vaginal acidity: high d/l lactate ratio is consistent with bacteria being the primary source. Hum Reprod 16:1809–1813. doi:10.1093/humrep/16.9.1809. 11527880

[21] Witkin SS, Mendes-Soares H, Linhares IM, Jayaram A, Ledger WJ, Forney LJ 2013 Influence of vaginal bacteria and d- and l-lactic acid isomers on vaginal extracellular matrix metalloproteinase inducer: implications for protection against upper genital tract infections. mBio 4:e00460-13. doi:10.1128/mBio.00460-13. 23919998 PMC3735189

[22] O’Hanlon DE, Moench TR, Cone RA 2013 Vaginal pH and microbicidal lactic acid when lactobacilli dominate the microbiota. PLoS One 8:e80074. doi:10.1371/journal.pone.0080074. 24223212 PMC3819307

[23] Gajer P, Brotman RM, Bai G, Sakamoto J, Schütte UM, Zhong X, Koenig SS, Fu L, Ma ZS, Zhou X, Abdo Z, Forney LJ, Ravel J 2012 Temporal dynamics of the human vaginal microbiota. Sci Transl Med 4:132ra52. doi:10.1126/scitranslmed.3003605. PMC3722878 22553250

[24] Al-Mushrif S, Eley A, Jones BM 2000 Inhibition of chemotaxis by organic acids from anaerobes may prevent a purulent response in bacterial vaginosis. J Med Microbiol 49:1023–1030. doi:10.1099/0022-1317-49-11-1023. 11073156

[25] Mirmonsef P, Gilbert D, Zariffard MR, Hamaker BR, Kaur A, Landay AL, Spear GT 2011 The effects of commensal bacteria on innate immune responses in the female genital tract. Am J Reprod Immunol 65:190–195. doi:10.1111/j.1600-0897.2010.00943.x. 21143335 PMC3581076

[26] Isaacs CE, Xu W 2013 Theaflavin-3,3′-digallate and lactic acid combinations reduce herpes simplex virus infectivity. Antimicrob Agents Chemother 57:3806–3814. doi:10.1128/AAC.00659-13. 23716050 PMC3719710

[27] Gong Z, Luna Y, Yu P, Fan H 2014 Lactobacilli inactivate Chlamydia trachomatis through lactic acid but not H_2_O_2_. PLoS One 9:e107758. doi:10.1371/journal.pone.0107758. 25215504 PMC4162611

[28] Nardini P, Ñahui Palomino RA, Parolin C, Laghi L, Foschi C, Cevenini R, Vitali B, Marangoni A 2016 Lactobacillus crispatus inhibits the infectivity of Chlamydia trachomatis elementary bodies, in vitro study. Sci Rep 6:29024. doi:10.1038/srep29024. 27354249 PMC4926251

[29] Graver MA, Wade JJ 2011 The role of acidification in the inhibition of Neisseria gonorrhoeae by vaginal lactobacilli during anaerobic growth. Ann Clin Microbiol Antimicrob 10:8. doi:10.1186/1476-0711-10-8. 21329492 PMC3045876

[30] O’Hanlon DE, Moench TR, Cone RA 2011 In vaginal fluid, bacteria associated with bacterial vaginosis can be suppressed with lactic acid but not hydrogen peroxide. BMC Infect Dis 11:200. doi:10.1186/1471-2334-11-200. 21771337 PMC3161885

[31] Aldunate M, Tyssen D, Johnson A, Zakir T, Sonza S, Moench T, Cone R, Tachedjian G 2013 Vaginal concentrations of lactic acid potently inactivate HIV. J Antimicrob Chemother 68:2015–2025. doi:10.1093/jac/dkt156. 23657804 PMC3743514

[32] Brittingham A, Wilson WA 2014 The antimicrobial effect of boric acid on Trichomonas vaginalis. Sex Transm Dis 41:718–722. doi:10.1097/OLQ.0000000000000203. 25581807

[33] Boskey ER, Moench TR, Hees PS, Cone RA 2003 A self-sampling method to obtain large volumes of undiluted cervicovaginal secretions. Sex Transm Dis 30:107–109. doi:10.1097/00007435-200302000-00002. 12567165

[34] Salazar-Gonzalez JF, Salazar MG, Keele BF, Learn GH, Giorgi EE, Li H, Decker JM, Wang S, Baalwa J, Kraus MH, Parrish NF, Shaw KS, Guffey MB, Bar KJ, Davis KL, Ochsenbauer-Jambor C, Kappes JC, Saag MS, Cohen MS, Mulenga J, Derdeyn CA, Allen S, Hunter E, Markowitz M, Hraber P, Perelson AS, Bhattacharya T, Haynes BF, Korber BT, Hahn BH, Shaw GM 2009 Genetic identity, biological phenotype, and evolutionary pathways of transmitted/founder viruses in acute and early HIV-1 infection. J Exp Med 206:1273–1289. doi:10.1084/jem.20090378. 19487424 PMC2715054

[35] Valore EV, Park CH, Igreti SL, Ganz T 2002 Antimicrobial components of vaginal fluid. Am J Obstet Gynecol 187:561–568. doi:10.1067/mob.2002.125280. 12237628

[36] Venkataraman N, Cole AL, Svoboda P, Pohl J, Cole AM 2005 Cationic polypeptides are required for anti-HIV-1 activity of human vaginal fluid. J Immunol 175:7560–7567. doi:10.4049/jimmunol.175.11.7560. 16301665

[37] Ravel J, Gajer P, Abdo Z, Schneider GM, Koenig SS, McCulle SL, Karlebach S, Gorle R, Russell J, Tacket CO, Brotman RM, Davis CC, Ault K, Peralta L, Forney LJ 2011 Vaginal microbiome of reproductive-age women. Proc Natl Acad Sci U S A 108(Suppl 1):4680–4687. doi:10.1073/pnas.1002611107. 20534435 PMC3063603

[38] Ravel J, Brotman RM 2016 Translating the vaginal microbiome: gaps and challenges. Genome Med 8:35. doi:10.1186/s13073-016-0291-2. 27036316 PMC4818402

[39] Brotman RM, Gajer P, Robinson CK, Ma B, Humphrys M, Tuddenham S, Ravel J, Ghanem KG 2015 Hormonal contraception is associated with stability and lactobacillus-dominance of the vaginal microbiota in a two-year observational study. Sex Transm Infect 91:A53. doi:10.1136/sextrans-2015-052270.148.

[40] Ñahui Palomino RA, Zicari S, Vanpouille C, Vitali B, Margolis L 2017 Vaginal Lactobacillus inhibits HIV-1 replication in human tissues ex vivo. Front Microbiol 8:906. doi:10.3389/fmicb.2017.00906. 28579980 PMC5437121

[41] Kyongo JK, Crucitti T, Menten J, Hardy L, Cools P, Michiels J, Delany-Moretlwe S, Mwaura M, Ndayisaba G, Joseph S, Fichorova R, van de Wijgert J, Vanham G, Ariën KK, Jespers V 2015 Cross-sectional analysis of selected genital tract immunological markers and molecular vaginal microbiota in sub-Saharan African women, with relevance to HIV risk and prevention. Clin Vaccine Immunol 22:526–538. doi:10.1128/CVI.00762-14. 25761460 PMC4412937

[42] Ghosh M, Fahey JV, Shen Z, Lahey T, Cu-Uvin S, Wu Z, Mayer K, Wright PF, Kappes JC, Ochsenbauer C, Wira CR 2010 Anti-HIV activity in cervical-vaginal secretions from HIV-positive and -negative women correlate with innate antimicrobial levels and IgG antibodies. PLoS One 5:e11366. doi:10.1371/journal.pone.0011366. 20614007 PMC2894072

[43] Tachedjian G, Aldunate M, Bradshaw CS, Cone RA 2017 The role of lactic acid production by probiotic Lactobacillus species in vaginal health. Res Microbiol 168:782–792. doi:10.1016/j.resmic.2017.04.001. 28435139

[44] O’Hanlon DE, Lanier BR, Moench TR, Cone RA 2010 Cervicovaginal fluid and semen block the microbicidal activity of hydrogen peroxide produced by vaginal lactobacilli. BMC Infect Dis 10:120. doi:10.1186/1471-2334-10-120. 20482854 PMC2887447

[45] Lai SK, Hida K, Shukair S, Wang YY, Figueiredo A, Cone R, Hope TJ, Hanes J 2009 Human immunodeficiency virus type 1 is trapped by acidic but not by neutralized human cervicovaginal mucus. J Virol 83:11196–11200. doi:10.1128/JVI.01899-08. 19692470 PMC2772788

[46] Ghosh M 2014 Secreted mucosal antimicrobials in the female reproductive tract that are important to consider for HIV prevention. Am J Reprod Immunol 71:575–588. doi:10.1111/aji.12250. 24754244

[47] Cole AM, Cole AL 2017 HIV-enhancing and HIV-inhibiting properties of cationic peptides and proteins. Viruses 9. doi:10.3390/v9050108. PMC5454421 28505117

[48] Ghosh M, Shen Z, Fahey JV, Cu-Uvin S, Mayer K, Wira CR 2010 Trappin-2/Elafin: a novel innate anti-human immunodeficiency virus-1 molecule of the human female reproductive tract. Immunology. 129:207–219. doi:10.1111/j.1365-2567.2009.03165.x. 19824918 PMC2814463

[49] Smith JA, Daniel R 2016 Human vaginal fluid contains exosomes that have an inhibitory effect on an early step of the HIV-1 life cycle. AIDS 30:2611–2616. doi:10.1097/QAD.0000000000001236. 27536982 PMC6141010

[50] Cone RA 2014 Vaginal microbiota and sexually transmitted infections that may influence transmission of cell-associated HIV. J Infect Dis 210(Suppl 3):S616–S621. doi:10.1093/infdis/jiu459. 25414415 PMC4303075

[51] Boskey ER, Telsch KM, Whaley KJ, Moench TR, Cone RA 1999 Acid production by vaginal flora in vitro is consistent with the rate and extent of vaginal acidification. Infect Immun 67:5170–5175. 10496892 10.1128/iai.67.10.5170-5175.1999 PMC96867

[52] Hu J, Gardner MB, Miller CJ 2000 Simian immunodeficiency virus rapidly penetrates the cervicovaginal mucosa after intravaginal inoculation and infects intraepithelial dendritic cells. J Virol 74:6087–6095. doi:10.1128/JVI.74.13.6087-6095.2000. 10846092 PMC112107

[53] Mirmonsef P, Gilbert D, Veazey RS, Wang J, Kendrick SR, Spear GT 2012 A comparison of lower genital tract glycogen and lactic acid levels in women and macaques: implications for HIV and SIV susceptibility. AIDS Res Hum Retroviruses 28:76–81. doi:10.1089/aid.2011.0071. 21595610 PMC3251838

[54] Daggett GJ Jr, Zhao C, Connor-Stroud F, Oviedo-Moreno P, Moon H, Cho MW, Moench T, Anderson DJ, Villinger F 2017 Comparison of the vaginal environment in rhesus and cynomolgus macaques pre- and post-lactobacillus colonization. J Med Primatol 46:232–238. doi:10.1111/jmp.12264. 28488364 PMC5597444

[55] Nunn KL, Wang YY, Harit D, Humphrys MS, Ma B, Cone R, Ravel J, Lai SK 2015 Enhanced trapping of HIV-1 by human cervicovaginal mucus is associated with Lactobacillus crispatus-dominant microbiota. mBio 6:e01084-15. doi:10.1128/mBio.01084-15. 26443453 PMC4611035

[56] Shukair SA, Allen SA, Cianci GC, Stieh DJ, Anderson MR, Baig SM, Gioia CJ, Spongberg EJ, Kauffman SM, McRaven MD, Lakougna HY, Hammond C, Kiser PF, Hope TJ 2013 Human cervicovaginal mucus contains an activity that hinders HIV-1 movement. Mucosal Immunol 6:427–434. doi:10.1038/mi.2012.87. 22990624 PMC3732193

[57] Olmsted SS, Khanna KV, Ng EM, Whitten ST, Johnson ON III, Markham RB, Cone RA, Moench TR 2005 Low pH immobilizes and kills human leukocytes and prevents transmission of cell-associated HIV in a mouse model. BMC Infect Dis 5:79. doi:10.1186/1471-2334-5-79. 16194280 PMC1262719

[58] Low N, Chersich MF, Schmidlin K, Egger M, Francis SC, van de Wijgert JH, Hayes RJ, Baeten JM, Brown J, Delany-Moretlwe S, Kaul R, McGrath N, Morrison C, Myer L, Temmerman M, van der Straten A, Watson-Jones D, Zwahlen M, Hilber AM 2011 Intravaginal practices, bacterial vaginosis, and HIV infection in women: individual participant data meta-analysis. PLoS Med 8:e1000416. doi:10.1371/journal.pmed.1000416. 21358808 PMC3039685

[59] Lai SK, O’Hanlon DE, Harrold S, Man ST, Wang YY, Cone R, Hanes J 2007 Rapid transport of large polymeric nanoparticles in fresh undiluted human mucus. Proc Natl Acad Sci U S A 104:1482–1487. doi:10.1073/pnas.0608611104. 17244708 PMC1785284

[60] Lai SK, Wang YY, Hida K, Cone R, Hanes J 2010 Nanoparticles reveal that human cervicovaginal mucus is riddled with pores larger than viruses. Proc Natl Acad Sci U S A 107:598–603. doi:10.1073/pnas.0911748107. 20018745 PMC2818964

[61] Sonza S, Maerz A, Deacon N, Meanger J, Mills J, Crowe S 1996 Human immunodeficiency virus type 1 replication is blocked prior to reverse transcription and integration in freshly isolated peripheral blood monocytes. J Virol 70:3863–3869. 8648722 10.1128/jvi.70.6.3863-3869.1996 PMC190263

[62] Gartner S, Markovits P, Markovitz DM, Kaplan MH, Gallo RC, Popovic M 1986 The role of mononuclear phagocytes in HTLV-III/LAV infection. Science 233:215–219. doi:10.1126/science.3014648. 3014648

[63] Wapling J, Moore KL, Sonza S, Mak J, Tachedjian G 2005 Mutations that abrogate human immunodeficiency virus type 1 reverse transcriptase dimerization affect maturation of the reverse transcriptase heterodimer. J Virol 79:10247–10257. doi:10.1128/JVI.79.16.10247-10257.2005. 16051818 PMC1182633

[64] Yap SH, Sheen CW, Fahey J, Zanin M, Tyssen D, Lima VD, Wynhoven B, Kuiper M, Sluis-Cremer N, Harrigan PR, Tachedjian G 2007 N348I in the connection domain of HIV-1 reverse transcriptase confers zidovudine and nevirapine resistance. PLoS Med 4:e335. doi:10.1371/journal.pmed.0040335. 18052601 PMC2100143

[65] Saladini F, Giannini A, Boccuto A, Vicenti I, Zazzi M 2018 Agreement between an in-house replication competent and a reference replication defective recombinant virus assay for measuring phenotypic resistance to HIV-1 protease, reverse transcriptase, and integrase inhibitors. J Clin Lab Anal 32. doi:10.1002/jcla.22206. PMC6816933 28303602

[66] Nugent RP, Krohn MA, Hillier SL 1991 Reliability of diagnosing bacterial vaginosis is improved by a standardized method of gram stain interpretation. J Clin Microbiol 29:297–301. 1706728 10.1128/jcm.29.2.297-301.1991 PMC269757

[67] Fadrosh DW, Ma B, Gajer P, Sengamalay N, Ott S, Brotman RM, Ravel J 2014 An improved dual-indexing approach for multiplexed 16S rRNA gene sequencing on the Illumina MiSeq platform. Microbiome 2:6. doi:10.1186/2049-2618-2-6. 24558975 PMC3940169

[68] United States Environmental Protection Agency 2000 Guidance for data quality assessment: practical methods for data analysis EPA QA/G-9 QA00 Update, p 20460 Office of Environmental Information, Washington, DC.

[69] Efron B 1979 Bootstrap methods: another look at the jackknife. Ann Statist 7:1–26. doi:10.1214/aos/1176344552.

[70] Huber P 1967 The behaviour of maximum likelihood estimates under nonstandard conditions, p 221–233. *In* Fifth Berkeley Symposium on Mathematical Statistics and Probability. University of California Press, Berkeley, CA.

