# Supplementary material for: Anti-HIV-1 Activity of Lactic Acid in Human Cervicovaginal Fluid
Source: mSphere. 2018 Jul 5;3(4):e00055-18. doi: 10.1128/mSphere.00055-18 (PMC6034077; doi:10.1128/mSphere.00055-18)
Supplement: TABLE S1 [file sph003182579st1.pdf]

Table S1. Donor Demographics

Participant ID[a]	CVF Sample ID[b]	Sample collection Date	Age	Race	Hormonal Contraceptive Use
1	HIV_2	Jan 2013	21	Black	No
2	HIV_3[c]	Jan 2013	22	White	Yes
3	HIV_4[c]	Jan 2013	24	White	No
4	HIV_5[c]	Jan 2013	27	Asian	No
2	HIV_7	Feb 2013	22	White	Yes
4	HIV_8[c]	Feb 2013	27	Asian	No
5	HIV_9	Feb 2013	24	White	No
6	HIV_11	Feb 2013	19	White	N/A
7	HIV_12	Feb 2013	22	White	N/A
8	HIV_13	Feb 2013	25	White	No
9	HIV_15	Jan 2014	22	White	Yes
10	HIV_16	Jan 2014	21	White	N/A
11	HIV_17	Jan 2014	28	Asian	Yes
3	HIV_18[c]	Jan 2014	25	White	No
12	HIV_19	Jan 2014	25	White	N/A
13	HIV_20	Jan 2014	28	White	N/A
14	HIV_21	Jan 2014	N/A[d]	N/A	N/A
15	HIV_22	Jan 2014	19	Asian	N/A
16	HIV_23	Jan 2014	23	White	N/A
17	HIV_24	Jan 2014	20	Other	N/A
18	HIV_25	Jan 2014	24	Asian	N/A
19	HIV_26	Jan 2014	21	N/A	N/A
20	HIV_27	Jan 2014	27	White	N/A

[a]Participant identification number
[b]Cervicovaginal fluid (CVF) sample identification number
[c]CVF from same donors collected on different days.
Samples HIV_3 and HIV_7 are from donor 2, HIV_4 and HIV_18 from donor 3, and HIV_5 and HIV_8 are from donor 4.
[d]Not available.
