# Supplementary material for: Anti-HIV-1 Activity of Lactic Acid in Human Cervicovaginal Fluid
Source: mSphere. 2018 Jul 5;3(4):e00055-18. doi: 10.1128/mSphere.00055-18 (PMC6034077; doi:10.1128/mSphere.00055-18)
Supplement: TABLE S2 [file sph003182579st2.docx]

**Table S2. Donor sample characterization**

| **CVF**  **Sample ID^a^** | **Nugent**  **Score^b^** | **CST^d^** | **pH (+HIV)^e^** | **%D-LA^f^** | **%L-LA^g^** | **%D+L-LA^h^** | **%D-LAH^i^** | **%L-LAH^i^** | **%D+L-LAH^i^** | **Ratio**  **D:L-LA^j^** | **Infectivity^k^** |
| --- | --- | --- | --- | --- | --- | --- | --- | --- | --- | --- | --- |
| HIV_2 | 0 | ND | 4.03 | 0.760 | 0.480 | 1.241 | 0.307 | 0.194 | 0.500 | 1.58 | 0.018 |
| HIV_3 | 0 | ND | 4.34 | 0.396 | 0.490 | 0.886 | 0.098 | 0.122 | 0.220 | 0.81 | -0.001 |
| HIV_4 | ND^c^ | ND | 4.20 | 0.399 | 0.200 | 0.599 | 0.125 | 0.063 | 0.188 | 1.98 | 0.008 |
| HIV_5 | 0 | ND | 4.00 | 0.122 | 0.302 | 0.423 | 0.051 | 0.127 | 0.178 | 0.40 | -0.001 |
| HIV_7 | 0 | ND | 4.36 | 0.424 | 0.308 | 0.732 | 0.102 | 0.074 | 0.176 | 1.38 | 0.140 |
| HIV_8 | 0 | ND | 3.97 | 0.566 | 0.413 | 0.980 | 0.248 | 0.181 | 0.428 | 1.37 | -0.001 |
| HIV_9 | 2 | ND | 4.18 | 0.466 | 0.575 | 1.041 | 0.151 | 0.186 | 0.337 | 0.81 | 0.031 |
| HIV_11 | 0 | ND | 4.02 | 0.035 | 0.291 | 0.326 | 0.014 | 0.119 | 0.133 | 0.12 | 0.034 |
| HIV_12 | 2 | ND | 5.43 | 0.663 | 0.443 | 1.106 | 0.017 | 0.012 | 0.029 | 1.50 | 1.345 |
| HIV_13 | 1 | ND | 4.01 | 0.524 | 0.403 | 0.927 | 0.217 | 0.167 | 0.384 | 1.30 | 0.029 |
| HIV_15 | 0 | CST I | 4.05 | 0.631 | 0.415 | 1.046 | 0.247 | 0.163 | 0.410 | 1.52 | -0.001 |
| HIV_16 | 0 | CST I | 4.00 | 0.636 | 0.443 | 1.079 | 0.267 | 0.186 | 0.453 | 1.44 | -0.001 |
| HIV_17 | 3 | CST III | 4.07 | 0.586 | 0.741 | 1.327 | 0.224 | 0.282 | 0.506 | 0.79 | -0.001 |
| HIV_18 | 2 | CST I | 4.15 | 0.750 | 0.365 | 1.115 | 0.254 | 0.124 | 0.378 | 2.05 | -0.001 |
| HIV_19 | 2 | CST I | 4.02 | 0.662 | 0.459 | 1.122 | 0.271 | 0.188 | 0.459 | 1.44 | -0.001 |
| HIV_20 | 0 | CST I | 4.07 | 0.618 | 0.349 | 0.967 | 0.236 | 0.133 | 0.369 | 1.77 | -0.001 |
| HIV_21 | 2 | CST I | 3.95 | 0.766 | 0.541 | 1.307 | 0.344 | 0.243 | 0.586 | 1.42 | -0.001 |
| HIV_22 | 0 | CST I | 4.07 | 0.843 | 0.394 | 1.237 | 0.322 | 0.150 | 0.472 | 2.14 | -0.001 |
| HIV_23 | 2 | CST I | 4.54 | 0.837 | 0.497 | 1.334 | 0.145 | 0.086 | 0.231 | 1.68 | 0.066 |
| HIV_24 | 0 | CST V | 4.21 | 0.686 | 0.513 | 1.199 | 0.212 | 0.158 | 0.370 | 1.34 | 0.004 |
| HIV_25 | 5 | CST V | 4.94 | 0.176 | 0.162 | 0.338 | 0.014 | 0.012 | 0.026 | 1.09 | 0.609 |
| HIV_26 | 0 | CST I | 4.26 | 0.724 | 0.316 | 1.040 | 0.206 | 0.090 | 0.296 | 2.29 | 0.008 |
| HIV_27 | 0 | CST V | 4.11 | 0.711 | 0.414 | 1.125 | 0.256 | 0.149 | 0.405 | 1.72 | -0.001 |

^a^ Cervicovaginal fluid (CVF) sample identification number

^b^ Normal (0 – 3), Intermediate (4 – 6), Bacterial vaginosis (7 – 10)

^c^ Not done

^d^ Community state type (CST), dominated by *L. crispatus* (CST I); *L.iners*-dominated but contains a substantial proportion of other species (CST-IIIB); *L. jensenii* dominated (CST V)

^e^ Final pH of CVF following addition of HIV-1 (5% volume of CVF)

^f^ Final concentration of D-isomer of lactic acid (LA) in CVF sample in % (w/v) following minimal dilution by addition of HIV-1.

^g^ Final concentration of L-isomer of lactic acid (LA) in CVF sample in % (w/v) following minimal dilution by addition of HIV-1.

^h^ Total concentration of D- and L-isomers of lactic acid (LA) in CVF sample in % (w/v) following minimal dilution by addition of HIV-1.

^i^ Final concentration of protonated D-LA, L-LA or D+L-LA in % (w/v) following minimal dilution by addition of HIV-1.

^j^ Ratio of D- to L-LA in sample

^k^ HIV_Ba-L_ infectivity normalised to DMEM-10
