# Supplementary figures and images for: Anti-HIV-1 Activity of Lactic Acid in Human Cervicovaginal Fluid
Source: mSphere. 2018 Jul 5;3(4):e00055-18. doi: 10.1128/mSphere.00055-18 (PMC6034077; doi:10.1128/mSphere.00055-18)

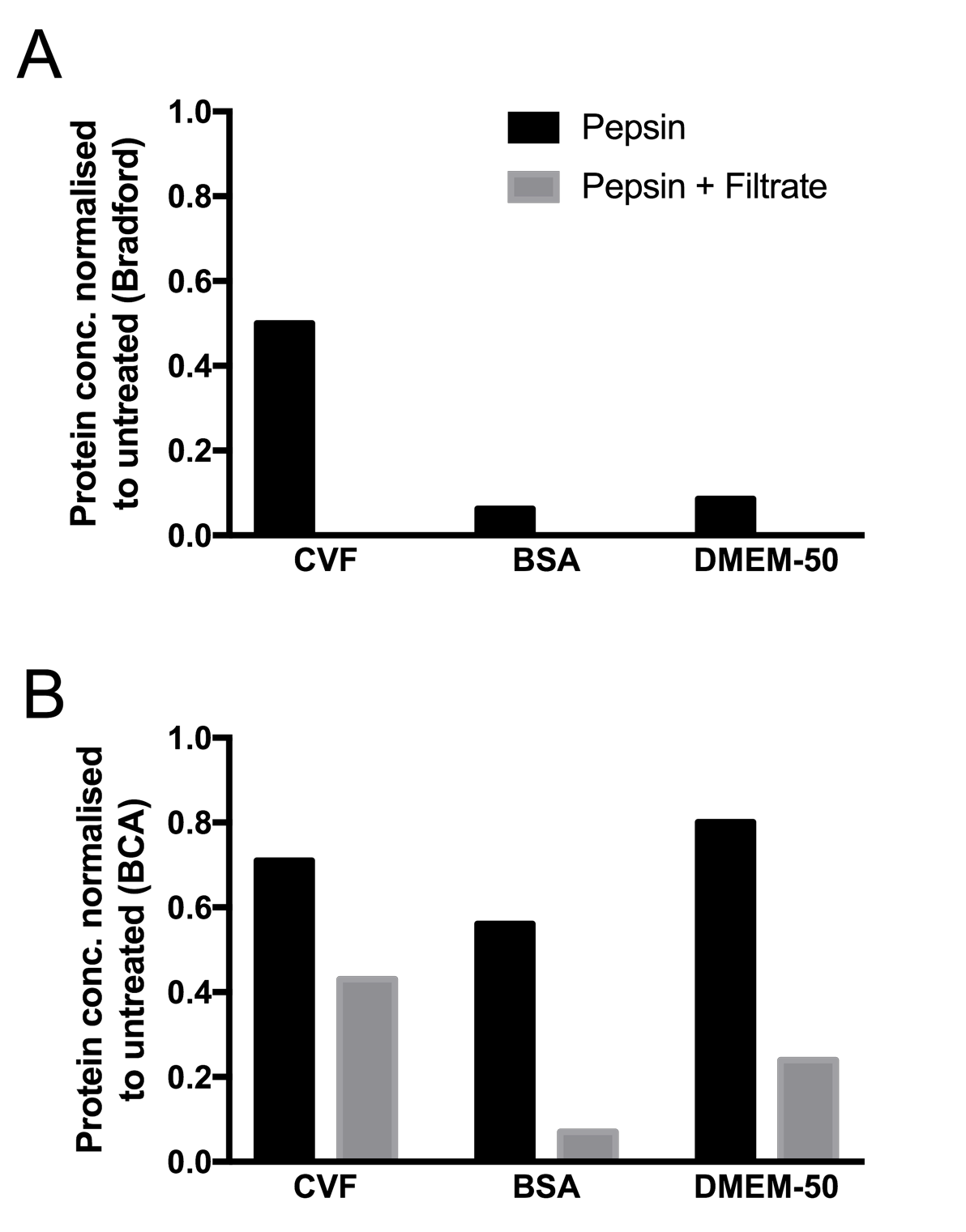

Supplement: FIG S1 [file sph003182579sf1.tif]
